# The Contribution of MicroRNAs to the Inflammatory and Neoplastic Characteristics of Erdheim–Chester Disease

**DOI:** 10.3390/cancers12113240

**Published:** 2020-11-03

**Authors:** Ran Weissman, Eli L. Diamond, Julien Haroche, Nir Pillar, Guy Shapira, Benjamin H. Durham, Justin Buthorn, Fleur Cohen, Michelle Ki, Galia Stemer, Gary A. Ulaner, Zahir Amoura, Jean-François Emile, Roei D. Mazor, Noam Shomron, Omar I. Abdel-Wahab, Ofer Shpilberg, Oshrat Hershkovitz-Rokah

**Affiliations:** 1 Department of Molecular Biology, Faculty of Natural Sciences, Ariel University, Ariel 40700, Israel; ran20088@gmail.com; 2 Translational Research Lab, Assuta Medical Centers, Tel-Aviv 6971028, Israel; ofers@assuta.co.il; 3 Department of Neurology, Memorial Sloan Kettering Cancer Center, New York, NY 10016, USA; diamone1@mskcc.org (E.L.D.); buthornj@mskcc.org (J.B.); 4 Service de Médecine Interne, Hôpital Universitaire Pitié Salpêtrière-Charles Foix, Sorbonne Université, Faculté de Médecine, 75013 Paris, France; julien.haroche@aphp.fr (J.H.); fleur.cohen@aphp.fr (F.C.); zahir.amoura@aphp.fr (Z.A.); 5 Department of Pathology, Hadassah Medical Center and Hebrew University, Jerusalem 91120, Israel; nirpillar@gmail.com; 6 Edmond J. Safra Center of Bioinformatics, Sackler Faculty of Medicine, Tel Aviv University, Tel Aviv 69978, Israel; guyspersonal@gmail.com (G.S.); nshomron@post.tau.ac.il (N.S.); 7 Human Oncology and Pathogenesis Program, Memorial Sloan Kettering Cancer Center, New York, NY 10016, USA; durhamb@mskcc.org (B.H.D.); mck96@cornell.edu (M.K.); abdelwao@mskcc.org (O.I.A.-W.); 8 Department of Pathology, Memorial Sloan Kettering Cancer Center, New York, NY 10016; USA; 9 HaEmek Medical Center, Department of Hematology, Afula 1834111, Israel; galia_st@clalit.org.il; 10 Department of Radiology, Memorial Sloan Kettering Cancer Center, New York, NY 10016, USA; Gary.Ulaner@hoag.org; 11 Research Unit EA4340, Versailles University, Paris-Saclay University, 92104 Boulogne, France; jean-francois.emile@uvsq.fr; 12 Pathology Department, Ambroise Paré Hospital, Assistance Publique-Hôpitaux de Paris (AP-HP), 92104 Boulogne, France; 13 Assuta Medical Centers, Institute of Hematology/Clinic of Histiocytic Neoplasms, Tel-Aviv 6971028, Israel; rd.mazor@gmail.com; 14 Department of Medicine, Adelson School of Medicine, Ariel University, Ariel 40700, Israel

**Keywords:** Erdheim–Chester disease, histiocytosis, microRNA, MAPK/ERK pathway

## Abstract

**Simple Summary:**

In the last two decades, new molecules, named microRNAs, have been identified. Impairment of microRNA function can lead to the development of diseases such as cancer; therefore, analyzing microRNAs expression, may help to explain the development of diseases. Moreover, these molecules can be obtained easily from blood, with little discomfort to the patient, and this may help to develop them as substances that could help to diagnose diseases and determine response to treatment. Here, we studied how microRNAs are involved in a rare clonal hematological malignancy named Erdheim–Chester disease (ECD). We found a differential expression microRNA signature between ECD patients and healthy controls. Our analysis suggests that reduced expression of microRNAs in ECD results in upregulation of target genes that participate in cell survival signaling and inflammation. This study expands our knowledge of the molecular basis of ECD and may enable improved treatment for affected patients.

**Abstract:**

The pathogenesis of histiocytic neoplasms is driven by mutations activating the MAPK/ERK pathway, but little is known about the transcriptional and post-transcriptional alterations involved in these neoplasms. We analyzed microRNA (miRNA) expression in plasma samples and tissue biopsies of Erdheim–Chester disease (ECD) and Langerhans cell histiocytosis (LCH) patients. In silico analysis revealed a potential role of miRNAs in regulating gene expression in these neoplasms as compared with healthy controls (HC). NanoString analysis revealed 101 differentially expressed plasma miRNAs in 16 ECD patients as compared with 11 HC, 95% of which were downregulated. MiRNAs-15a-5p, -15b-5p, -21-5p, -107, -221-3p, -320e, -630, and let-7 family miRNAs were further evaluated by qRT-PCR in an extended cohort of 32 ECD patients, seven LCH and 15 HC. Six miRNAs (let-7a, let-7c, miR-15a-5p, miR-15b-5p, miR-107 and miR-630) were highly expressed in LCH plasma and tissue samples as compared with ECD. Pathway enrichment analysis indicated the miRNA contribution to inflammatory and pro-survival signaling pathways. Moreover, the let-7 family members were downregulated in untreated ECD patients as compared with HC, while treatment with MAPK/ERK signaling inhibitors for 16 weeks resulted in their upregulation, which was in parallel with the radiologic response seen by PET-CT. The study highlights the potential contribution of miRNA to the inflammatory and neoplastic characteristics of ECD and LCH.

## 1. Introduction

Erdheim–Chester disease (ECD) is a rare hematological neoplasm that is histologically characterized by proliferation of CD68^+^, CD1a^−^ histiocytes in a background of inflammatory stroma. ECD has various manifestations, ranging from an asymptomatic disease to a disseminated, multi-systemic life-threatening entity, particularly when the heart or central nervous system are involved [1].

ECD is a disease characterized by cytokine perturbations [2,3] and symptomatology related to uncontrolled systemic inflammation [1,4]. The pathological cells express markers of the macrophage lineage, such as CD14 and CD68, and in the majority of cases stain negative for markers of dendritic lineage, such as CD1a and S-100 [5]. Such histological and immunohistochemical features help distinguish ECD from Langerhans cell histiocytosis (LCH), a disease that shares many similarities with ECD [6]. LCH is characterized by pathogenic dendritic cell accumulation in inflammatory lesions. Clinical presentations are highly variable, ranging from single lesions to potentially lethal disseminated disease [7]. 

The MAPK signaling pathway is well known for its role in governing cellular proliferation, differentiation, and apoptosis [8]. Particularly, aberrant signal transduction through the RAS/RAF/MEK/ERK axis has been repeatedly implicated in a multitude of malignancies, including melanoma [9], non-small cell lung carcinoma [10], colorectal cancer [11], papillary thyroid cancer [12], glioma [13], and hematological malignancies [14,15,16,17].

As RAF is the downstream effector of RAS, it is dependent on the interaction with an activated RAS in the healthy cellular state. The RAF family includes several variants (e.g., ARAF, BRAF, and CRAF) [18], all of which are serine/threonine kinases responsible for pathway progression via activation of MAP kinase (MEK 1/2) and extracellular signal-regulated kinases (ERK1/2). The phosphorylated targets of both MEK 1/2 and ERK1/2 are involved in a wide range of processes such as cell survival, proliferation, and differentiation. ERK1/2 has various phosphorylation targets, independent of cellular location and compartment. In the nucleus, ERK1/2 can activate transcription factors that lead to cell proliferation, making it an important antitumor target [18]. As such, small molecule-mediated targeted inhibition of the ERK signaling cascade is both actively investigated and clinically recognized as an acceptable therapeutic strategy in several cancers [9,19,20,21,22].

More than 50% of ECD and LCH patients harbor BRAF V600E mutations [23]. BRAF V600 wild-type ECD lesions have been found to nearly invariably harbor somatic mutations in the MAPK/ERK signaling pathway. A series of recurrent activating kinase mutations and fusions involving the ERK cascade and PI3K/AKT pathways have been discovered in a large proportion of ECD patients (reviewed in [1]). Specifically, nine activating MAP2K1 mutations were discovered in 50% (nine of 18) of BRAF wild-type archived ECD cases evaluated by targeted sequencing [24]. These MAP2K1 mutations caused constitutive ERK activation in vitro [24]. MAP2K1 mutations are not exclusive to histiocytosis and have been observed in other hematopoietic neoplasms. Other mutations in ECD include activating mutations in NRAS (Q61R), KRAS (G12S) [25,26,27], and PI3K/AKT pathway alterations (PI3KCA mutations) [24,25]. As a consequence, G12 and G13 mutants trap RAS in a constitutively active state. Mutations at Q61 inhibit intrinsic GTP hydrolysis and GAP-mediated GTP hydrolysis [28]. Mutations in ARAF were found in 21% (three of 14) of ECD specimens with two of them being mutually exclusive to BRAF V600E [24].

While genetic alterations driving ECD and LCH pathogenesis have been well studied [1,29,30,31], to date, only a few studies, have investigated transcriptional and post-transcriptional alterations in these diseases. 

Non-coding RNAs are a diverse family of evolutionarily conserved species that do not code for proteins and include long non-coding RNAs (lncRNAs), circular RNAs (circRNAs), and microRNAs (miRNAs), among others [32,33]. Much effort has been focused on the characterization of non-coding RNAs. Early work has shown that these species, particularly miRNAs, are central to cancer initiation, progression, and treatment response [34] via regulation of post-transcriptional gene expression. 

Chromosomal regions encompassing miRNAs involved in the negative regulation transcripts encoding known tumor suppressor genes can be amplified in cancer development. This amplification results in increased miRNA expression, consequently silencing the tumor suppressor gene. Conversely, miRNAs repressing oncogenes are often located in fragile loci, where deletions or mutations may lead to reduced miRNA levels and overexpression of the target oncogene [35]. By controlling the expression levels of their targets, several miRNAs have been shown to modulate the function of endothelial cells, [36]), dendritic cells [37], vascular smooth muscle cells [38], and macrophages [39,40,41]. Other studies have revealed that miRNAs control innate immunity [42], and abnormal expression of miRNAs has been associated with multiple immune disorders (reviewed in [43]). Moreover, genome-wide profiling has shown that miRNA expression signatures (miRNome) accurately discriminate among different types of cancer and are able to identify the tissue of origin of poorly differentiated tumors [44,45]. 

Patients with hematological malignancies have distinct miRNA expression profiles in plasma, serum, and tissues as compared to those of healthy individuals [46,47,48]; however, studies on non-coding RNA in histiocytic neoplasms are lacking. One of the major discoveries in the field of hematology is the miR-15/16 cluster which represents the most frequently deregulated miRNAs reported in chronic lymphocytic leukemia (CLL). These miRNAs have been associated with disease progression, prognosis, and drug resistance [45]. Nearly two-thirds of CLL cases show miR-15a/16-1 downregulation due to their location in the 13q14.3 locus, a genomic region frequently deleted in CLL patients [49]. MiR-15/16 functions as tumor suppressor by directly targeting BCL2. Thus, the loss of two negative regulators of BCL2 expression results in overexpression of BCL2. These findings led to the development of a specific BCL2 inhibitor (Venetoclax, AbbVie Inc., Chicago, IL, USA) that has now approved by the FDA for CLL, small lymphocytic lymphoma (SLL) and acute myeloid leukemia (AML) treatment. 

Overall, elucidating miRNAs molecular mechanism may contribute to determine more therapeutic targets for the treatment of histiocytosis patients. Therefore, we set out to evaluate the potential for dysregulated expression of miRNAs in ECD and LCH patients as compared with miRNA expression in healthy individuals and to discern their potential contribution to the pathogenesis of histiocytosis.

## 2. Results

### 2.1. MicroRNA Expression Patterns in Erdheim–Chester Disease (ECD) Patients as Compared with Healthy Individuals

Plasma samples were collected from 15 healthy volunteers who served as controls (median age 41 years, range 22–58), 32 untreated ECD patients (median age 56 years, range 18–83), and seven untreated LCH patients (median age 40 years, range 33–58) (Table 1). Lesions extracted from ECD (*n* = 5) and LCH (*n* = 4) patients are presented in Table 2.

**Figure 1:**
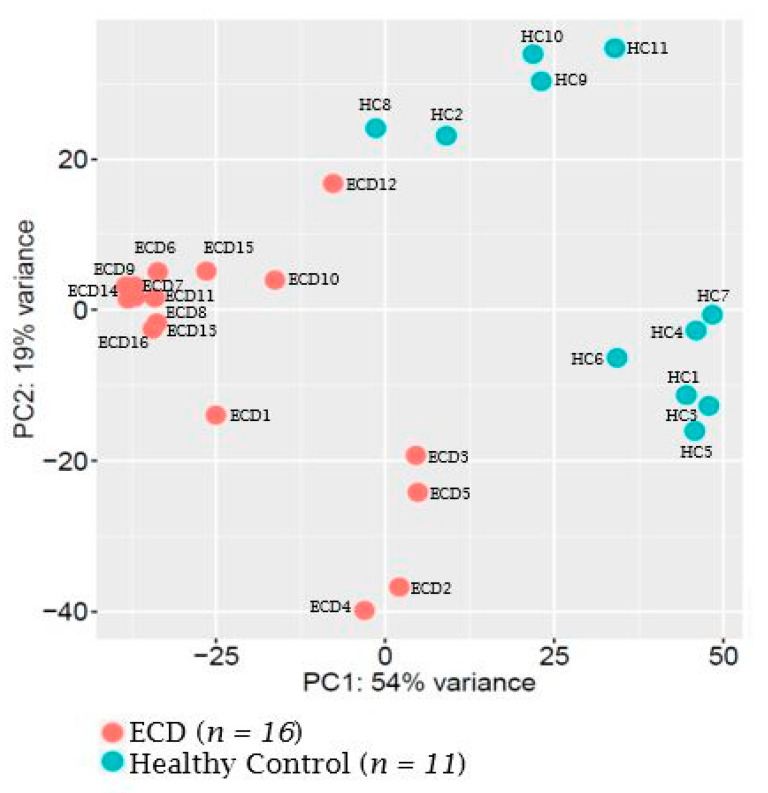
Differential microRNA expression in plasma samples of ECD patients and healthy controls (HC). Principal component analysis (PCA) mapping of NanoString nCounter Human miRNA expression assay. PCA analysis confirmed a differential expression pattern between plasma samples of ECD patients (red) and HC (blue).

The expression of 800 known mature human miRNAs in plasma was initially analyzed and compared between untreated, genetically annotated ECD patients (*n* = 16), and healthy controls (HC) (*n* = 11), using the NanoString nCounter Human miRNA expression assay. Due to insufficient biological material, we were not able to analyze the LCH samples by the NanoString assay. Principal component analysis (PCA) conducted for dimensionality reduction showed that ECD patients’ miRNAs were clustered separately from those of HC, indicating a distinct miRNA expression pattern (Figure 1).

**Figure 2:**
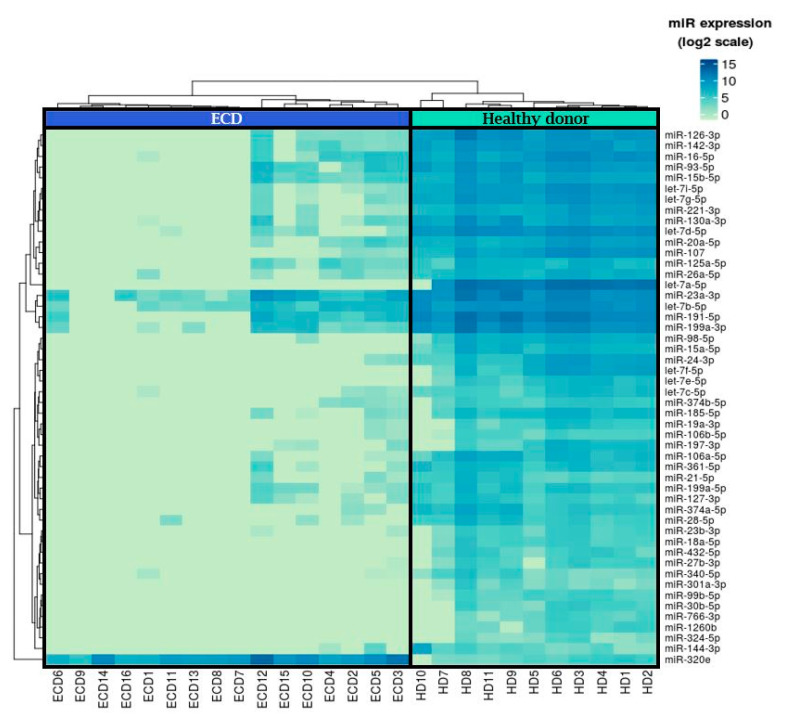
A heat map illustrating supervised clustering of the top 50 statistically significant miRNAs that were differentially expressed in samples from ECD patients as compared with samples from HC. Blue and green indicate the relatively high- and low-fold changes in miRNA expression, respectively.

Of the 800 miRNAs analyzed, 223 were expressed with a greater than two-fold change in plasma samples of ECD patients and HC. Of these, 101 miRNAs were significantly differentially expressed (96 were downregulated and five were upregulated) in ECD as compared with HC (FDR < 0.05) and included in the final analysis (Tables S1 and S2). Among the 50 miRNAs with the largest statistically significant difference between ECD and HC, 49 were downregulated and one was upregulated (Figure 2).

**Figure 3:**
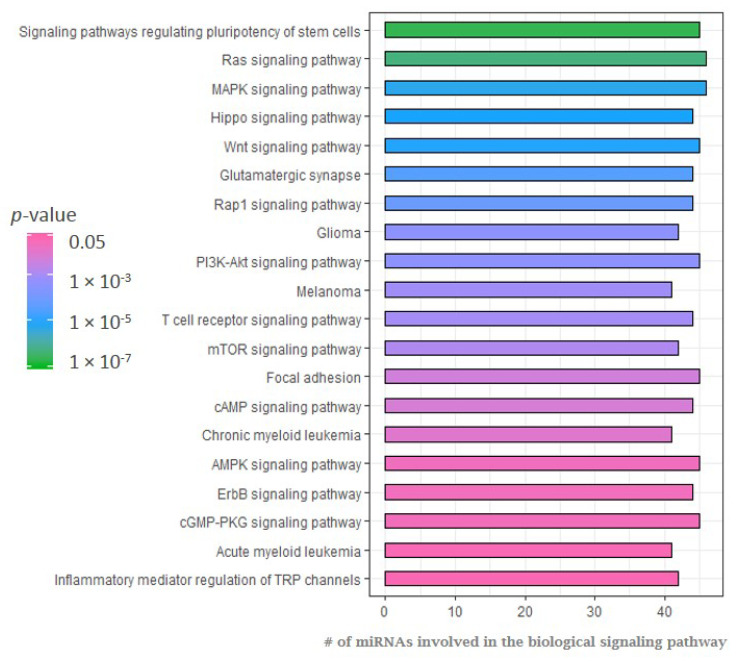
Enriched KEGG signaling pathways for the downregulated miRNAs represented in Figure 2 ordered by *p*-values (low to high). Signaling pathways were identified by the web-based computational tool DIANA-miRPath [50].

To detect miRNA gene targets and pathways implicated in ECD, in silico analysis of putative interactions among the top statistically significant 50 aberrantly expressed miRNAs and common signaling pathways was conducted using the web-based computational tool DIANA-miRPath. This computational tool estimates the impact of co-expressed miRNAs in biological pathways [50]. Pathway enrichment analysis in ECD patient interactions showed that the most statically significant downregulated miRNAs were enriched, among other pathways, for proliferation and inflammation signaling pathways (Figure 3 and Table S3), suggesting that reduced expression of miRNAs in ECD results in upregulation of target genes that participate in cell survival signaling and inflammation. A full list of enriched pathways is shown in the Figure S1.

Two of the most significantly enriched pathways were the Ras and MAPK/ERK signaling pathways (*p* = 5.05 × 10^−7^ and *p* = 5.26 × 10^−6^, respectively).

**Figure 4:**
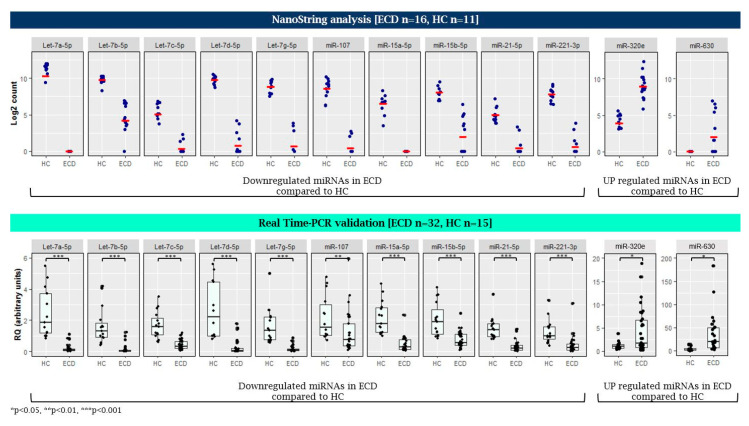
Validation of miRNAs expression in plasma samples of ECD patients and healthy controls (HC) by qRT-PCR (lower panel) following NanoString analysis (upper panel). qRT-PCR analysis was normalized to spike-in control cel-miR-39. RQ, relative quantification. * *p* < 0.05, ** *p* < 0.01, and *** *p* < 0.001.

To validate the NanoString results and to ensure that the variability observed is not technical, TaqMan qRT-PCR was performed on a subset of miRNAs that were differentially expressed in ECD as compared with HC. These included 10 downregulated miRNAs (let-7a-5p, let-7b-5p, let-7c-5p, let-7d-5p, let-7g-5p, miR-15a-5p, miR-15b-5p, miR-21-5p, miR-107, and miR-221-3p) and two upregulated miRNAs (miR-320e and miR-630) that were significantly different between ECD and HC (*p* < 9.3 × 10^−6^, log2 fold change >4.5) and were previously shown to have a role in inflammatory and neoplastic conditions [34,43], and therefore, may play a role in ECD. This analysis was done on a larger number of plasma samples which included 32 untreated ECD patients and 15 HC. The results obtained in the qRT-PCR analysis were similar to those obtained by the NanoString array (Figure 4). No significant correlation was found between the miRNA expression to the age of each group (*p* > 0.05).

### 2.2. Analysis of miRNA Expression in ECD Patients by Mutation Type

**Figure 5:**
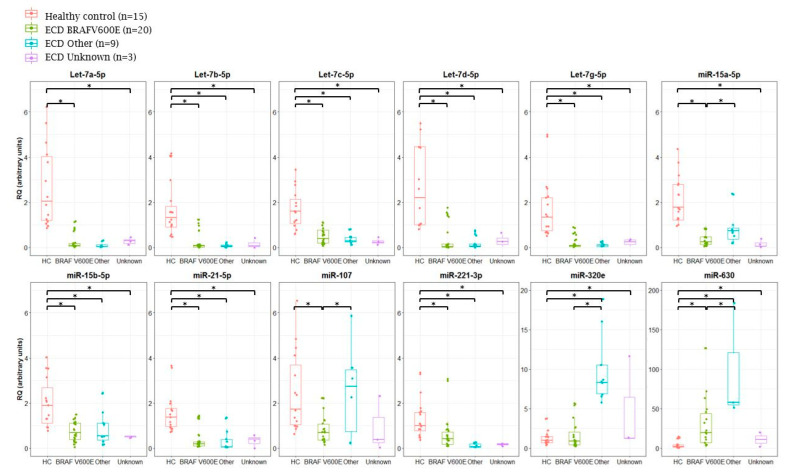
Analysis of miRNA expression in ECD patients by mutation type. MiRNA expression in ECD patient’s plasma samples as compared with plasma samples from healthy controls (HC) subjected to mutation analysis. “Other mutations” includes BRAF deletion and NRAS, KRAS, MAP2K2, and MAP2K1. * *p* < 0.05.

ECD patients were also analyzed by subgroup analysis by the following genetic mutations type: BRAF V600E mutation (*n* = 20); other mutations, i.e., BRAF deletion, NRAS, KRAS, MAP2K2, and MAP2K1 mutations (*n* = 9); and unknown mutations, which included patients who did not undergo next generation sequencing (NGS) due to insufficient biological material (*n* = 1) or no mutation was identified using the NGS panel (*n* = 2). Most miRNAs showed similar expression patterns among the various mutation types (Figure 5). However, miRNAs-107, -630, and -320e were more highly expressed in patients with other mutations as compared with the BRAF V600E mutation. (Patients with mutation in NRAS (ECD13); KRAS (ECD14, ECD19, ECD22); MAP2K2 (ECD15); MAP2K1 (ECD16, ECD20, ECD25); and BRAF deletion (ECD24) had elevated expression levels of miRNAs 320e and 630. Patients with mutations in MAP2K1 (ECD16, ECD20, ECD25) and one patient with unidentified mutation (ECD23) had elevated expression levels of miR-107) (Figure 5).

### 2.3. MiRNA Expression Patterns in Langerhans Cell Histiocytosis (LCH) Patients as Compared with ECD Patients and Healthy Individuals

**Figure 6:**
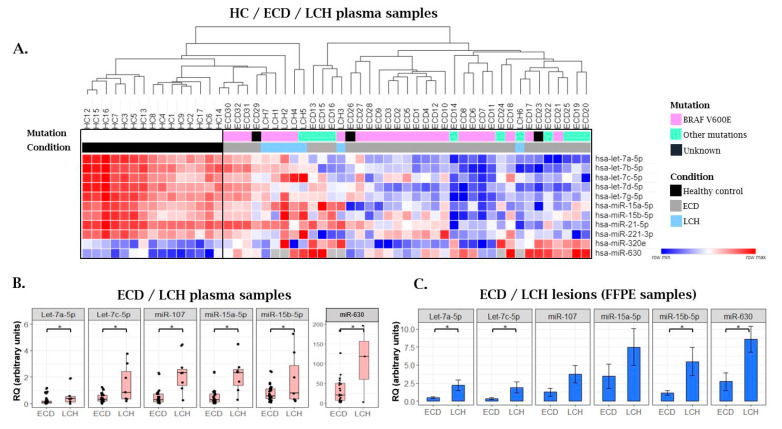
(**A**) A heat map illustrating supervised clustering of a subset of miRNAs that were differentially expressed in samples from ECD and LCH patients as compared with samples from healthy controls (HC). At each row, red and blue indicate the relatively high and low miRNA expression, respectively, as measured by qRT-PCR. Missing values are indicated in gray. Due to the lack of biological material, miR-630 was evaluated in 3 LCH plasma samples and not 7 plasma samples. “Other mutations” include BRAF deletion and NRAS, KRAS, MAP2K2, and MAP2K1; (**B**) Six miRNAs that were significantly higher in plasma samples of LCH patients as compared with ECD were analyzed in; (**C**) ECD (*n* = 5); and LCH (*n* = 4) lesions measured by qRT-PCR, normalized to spike-in control cel-miR-39. RQ, relative quantification. Bars represent low and high expression of the miRNA + SEM.

Next, we evaluated, by qRT-PCR, the miRNA expression in plasma samples of seven untreated LCH patients (Figure 6). Six miRNAs (let-7a-5p, let-7c-5p, miR-15a-5p, miR-15b-5p, miR-107, and miR-630) were significantly more highly expressed in plasma samples of LCH patients as compared with that of ECD patients (Figure 6B and Figure S2). Those miRNAs were successfully validated in ECD (*n* = 5) and LCH (*n* = 4) archived formalin fixed paraffin embedded (FFPE) tissue lesions and showed similar results with the expression levels of the plasma miRNAs expression (Figure 6C).

The expression of five plasma miRNAs (let-7a-5p, let-7b-5p, let-7d-5p, let-7g-5p, and miR-21-5p) was significantly lower in LCH as compared with HC. MiR-630 was upregulated in LCH patients as compared with ECD patients and HC (Figure 6A and Figure S2).

### 2.4. MiRNA Expression Following MAP/-ERK Signaling Cascade Inhibition

**Figure 7:**
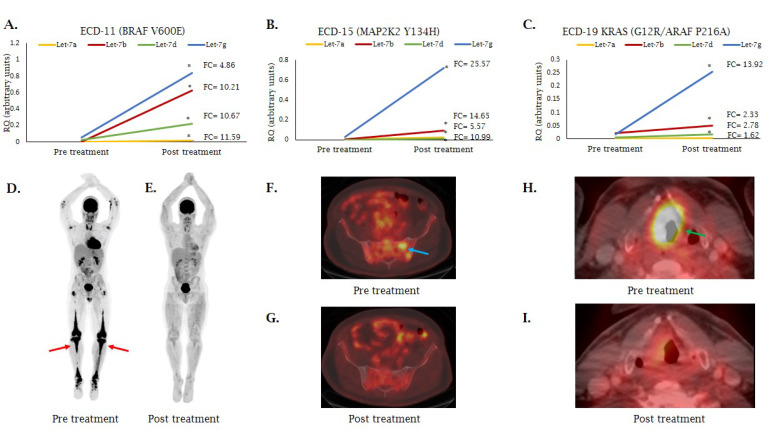
(**A**–**C**) MiRNA expression before and after treatment with the MEK inhibitor (cobimetinib). The graph shows the upregulation of let-7a-5p, let-7b-5p, let-7d-5p, and let-7g-5p after treatment with MEK inhibitor for 16 weeks. MiRNA expression was measured by qRT-PCR, normalized to spike-in control cel-miR-39. Fold change is shown on the right side of the line. * *p* < 0.05; (**D**) Full body maximal intensity projection (MIP) of FDG-PET demonstrates osseous disease, predominantly in the lower extremities, (**E**) that is resolved after four cycles of treatment with cobimetinib; (**F**) Axial fused FDG-PET/CT images demonstrate hypermetabolic iliac and sacral ECD lesions, (**G**) that are resolved after treatment. (**H**) Axial fused FDG-PET/CT images demonstrate hypermetabolic ECD lesions in the larynx, (**I**) that are resolved after treatment, with increased patency of the airway.

As mentioned above, the Ras and MAPK/ERK signaling pathways were found to be among the most significantly enriched pathways in our analysis of miRNA gene targets and putative gene network interactions in ECD (Figure 3). Our analysis also showed that the expression of let-7a-5p, let-7b-5p, let-7c-5p, let-7d-5p, and let-7g-5p was downregulated in untreated ECD patients as compared with HC. Since the MAPK/ERK pathway activation has been previously shown to enhance LIN28a stabilization, which in turn reduced the expression of the let-7 family miRNAs [51], we analyzed the expression of let-7a-5p, let-7b-5p, let-7c-5p, let-7d-5p, and let-7g-5p in eight ECD patients who were effectively treated with a MEK inhibitor (cobimetinib) and one patient (ECD28) treated with BRAF inhibitor (vemurafenib). After 16 weeks of treatment, let-7a-5p, let-7b-5p, let-7d-5p, and let-7g-5p showed upregulation in all nine patients (Figure 7A–C and Figure S3). This upregulation was in parallel to the response assessment by positron emission tomography computed tomography (PET-CT) (Figure 7D–I). Let-7c-5p levels were modestly elevated in five of nine patients with lower fold change as compared with the other family members (data not shown).

## 3. Discussion

In this study, we evaluated the expression profile of miRNAs in histiocytosis neoplasms. Analysis of the differential expression of miRNAs in plasma samples of ECD patients as compared with HC showed that the majority of differentially expressed miRNAs in ECD were downregulated. This finding is in line with previous reports, which showed that many cancers are characterized by miRNA downregulation [52,53].

Our analysis has been focused on 10 downregulated and two upregulated miRNAs. Five of the downregulated miRNAs belong to the let-7 family. This miRNA family is highly conserved among species, from nematode to human, suggesting it has a major function in gene expression regulation. Humans have multiple isoforms of let-7 miRNAs comprising nine mature let-7 miRNAs encoded by 12 different genomic loci, some of which are clustered together [54]. Since the different members of the let-7 family have similar seed sequences, they likely have overlapping sets of target mRNAs. However, these different members may have different functions depending on the cellular context of their expression. Many tumor types exhibit downregulation of let-7 family members’ expression [55]. One molecular mechanism is through the LIN28 family of small proteins which bind to let-7 family miRNAs and block their processing by Dicer ribonuclease, resulting in low levels of mature let-7 in undifferentiated cells (both normal embryonic stem cells and cancer cells) [56]. Iliopoulos et al. showed that overexpression of LIN28b upon the activation of NF-κB inhibited the generation of let-7 family miRNAs and increased the expression of IL-6, a target of let-7. In turn, IL-6 activated NF-κB and STAT3 transcription factors through the receptor tyrosine kinase (RTK) signaling pathway [57]. IL-6 is a cytokine that is highly expressed by ECD histiocytes. It is produced by macrophages and promotes activation and differentiation of T lymphocytes and macrophages. The cytokine is also involved in osteoclast differentiation and bone resorption [58]. Elevated serum levels were reported in ECD, in association with biochemical markers of bone turnover [59]. Let-7 is also a direct negative regulator of the Ras gene family [60]. Our results suggest that let-7 involvement is at an intersection between inflammation and cell transformation.

Since the MAPK/ERK pathway activation has previously been shown to enhance LIN28 stabilization [61], which in turn reduces the expression of let-7 family miRNAs as mentioned above, we examined the expression of let-7 family members (let-7a-5p, let-7b-5p, let-7c-5p, let-7d-5p, and let-7g-5p) in eight ECD patients treated with a MEK inhibitor and one patient treated with a BRAF inhibitor. Let-7 miRNAs were upregulated after 16 weeks of treatment in all treated patients and were in parallel to the evaluation of clinical response by PET-CT, suggesting that miRNAs from peripheral blood may be used as a simple monitoring tool for treatment response. Clinical monitoring by imaging technology, such as CT, PET/CT, or MRI, is widely used to determine treatment response in histiocytosis neoplasms. These technologies require high levels of expertise and some of them are associated with increased levels of irradiation. Therefore, new, reliable, and relatively non-invasive biomarkers with high sensitivity and specificity are necessary to improve diagnosis and prognosis of these diseases. However, additional samples from ECD patients at various time points during and after treatment with MAPK/ERK pathway inhibitors are required to determine whether let-7 upregulation is consistent over longer periods. It is important to note that all the patients responded to MAPK/ERK pathway inhibitor treatment and we do not have data on non-responders. Therefore, our observation that let-7 family miRNAs are upregulated following treatment with MEK inhibitors in parallel to the response seen by PET is only descriptive and should be further tested when treatment resistance will occur. In line with our observation of let-7 upregulation after treatment, Couts et al. showed that two let-7 family members (let-7g and let-7i) were upregulated in melanoma cells after treatment with a MEK inhibitor [62]. Moreover, several studies have suggested that the expression of let-7 family members in plasma or serum may be used as a diagnostic marker for several malignancies [55,63,64,65,66,67,68,69]. This suggest that, let-7 family miRNA expression may be used as biomarkers for treatment response in other malignancies.

One of the significantly enriched pathways suggested to be regulated in ECD by miRNAs in our analysis is the mammalian target of rapamycin (mTOR) signaling pathway, which regulates cell growth, proliferation, apoptosis, and modulates immune responses [70]. Gianfreda et al. demonstrated mTOR pathway activation in ECD lesions and provided preliminary evidence of the efficacy of a sirolimus (mTOR inhibitor) and prednisone-based regimen in ECD patients [71,72]. At our analysis, out of the 96 downregulated miRNAs in ECD patients, 42 miRNAs are predicated to be involved in the regulation of the mTOR pathway (Table S3), suggesting that these miRNAs may contribute to the upregulation of this pathway, specifically miRNAs-15a-5p, 15b-5p, 107, and 21-5p and the let-7 family miRNAs that were validated by qRT-PCR.

Similar to the let-7 family, the other miRNAs that were identified in the current study as differentially downregulated in ECD (miR-15a-5p, miR-15b-5p, miR-21-5p, miR-107, and miR-221-3p) are also known to be involved in inflammation and proliferation. The miRNA-15 family comprises six highly conserved miRNAs (miR-15a, miR-15b, miR-16-1, miR-16-2, miR-195, and miR-497). Chen et al. reported that restoration of miR-15a-5p expression in chronic myeloid leukemia (CML) cells decreased cell growth, metastasis, and enhanced cell apoptosis by targeting chemokine ligand 10 (CXCL10, also named interferon-γ inducible protein-10 (IP-10)) [73]. This member of the chemokine family has an important role in attracting and activating T cells, B cells, mononuclear macrophage, dendritic cells, NK cells, and other immune inflammatory cells [74,75]. ECD histiocytes were shown to express significantly higher levels of CXCL10 [3,76,77], suggesting a possible role for miRNA–mRNA regulation in these patients. Furthermore, miR-15a-5p also regulates VEGFA mRNA following inflammation and fibrosis in peritoneal mesothelial cells. Restoration of miR-15a-5p restrained inflammation and fibrosis of human peritoneal mesothelial cells by the TGF-β1/Smad2 signaling pathway [78].

An additional miRNA-15 family member found to be downregulated in ECD is miR-15b-5p. One of the verified targets of miR-15b-5p is SMAD7, which is a well-recognized inhibitor of osteoblast differentiation [79,80]. As the majority of ECD patients have bone involvement, miR-15b-5p might be an important target to promote osteoblast differentiation and prevent bone loss and fragility. Therefore, our findings suggest that miR-15 family members may have a role in the regulation of ECD pathogenesis.

MiR-21-5p targets IL-6, whereas miR-21 knockout mice have significantly higher levels of inflammatory cytokines, including IL-1β, IL-6, and TNF-α in cardiac tissues, as well as infiltration of CD11b^+^ monocytes/macrophages. Mechanistically, miR-21 deficiency enhanced p38 and NF-κB signaling activation, while miR-21 overexpression markedly inhibited inflammatory cytokine production [81].

MiR-107, a highly conserved miRNA that maps to intron 5 of the *PANK1* gene, contributes to the regulation of normal and tumor biological processes. There is limited information on miR-107 in hematological cancers. MiR-107 has been reported to be downregulated in acute promyelocytic leukemia (APL) blasts as compared with normal promyelocytes differentiated in vitro from CD34+ progenitors, and its expression level was upregulated after cells had been treated with ATRA [82]. A different study assessed global miRNA expression between purified B cells from treatment-naïve CLL patients and healthy controls and found that miR-107 was downregulated in CLL patients [83]. A later study showed that miR-107 targets calcium-channel protein (Cacna2d1) to promote erythroid differentiation in CML [84]. The strongest evidence that miR-107 is a tumor suppressor comes from studies on glioma; miR-107 expression was significantly downregulated in human glioma tissue and cell lines as compared with normal brain tissue. Additionally, low miR-107 expression was significantly associated with advanced pathological features and poor prognosis of human gliomas, such as larger tumor size, lower Karnofsky performance score, and shorter overall survival and progression-free survival [85]. Upregulation of this miR inhibited HMGB1, RAGE, MAPK, and NF-κB signaling pathways, and consequently suppressed expression of proinflammatory cytokines and matrix metalloproteinases [86]. Further studies are needed to elucidate the role of miR-107 in ECD.

MiR-320e and miR-630 levels were significantly elevated in ECD patients as compared with HC. MiR-320e was reported to be significantly elevated in patients with recurrent stage III colon cancer harboring the same V600E BRAF mutation, as in ECD [87].

MAP kinase phosphatases, also known as dual-specificity phosphatases (DUSPs), dephosphorylate many key signaling molecules including those in the MAPK/ERK cascade, leading to deactivation of the ERK pathway. Hence, DUSPs need to be properly controlled [88,89]. In silico analysis by miRDB [90] and TargetScan [91] bioinformatics tools showed that miR-320e is predicted to target several DUSP family members (DUSP2, DUSP4, DUSP14, DUSP18, DUSP19, DUSP22, and DUSP28), resulting in lower phosphatase activity and high ERK activation. Similarly, miR-630, which is also upregulated in ECD patients, is predicted to target DUSP4, DUSP6, and DUSP19. These findings should be further validated. To the best of our knowledge, the involvement of miR-630 in hematological malignancies has not been reported to date; however, high levels of this miRNA were found in renal cell carcinoma and were correlated with lower overall survival [92]. In ovarian cancer, high levels of miR-630 were shown to promote proliferation and migration of malignant cells [93]. Further investigation is needed to confirm if inhibition of these miRNAs may be beneficial for inhibiting ECD cell proliferation.

Comparison of miRNAs expression among ECD, LCH, and HC showed that let-7a-5p, let-7b-5p, let-7d-5p, let-7g-5p, and miR-21-5p were downregulated in both ECD and LCH as compared with HC. Conversely, let-7c-5p, miR-15a-5p, miR-15b-5p, and miR-107 expression were similar in LCH and HC, suggesting a different role for these miRNAs in each of the two histiocytosis entities that should be further examined. In addition, these miRNAs may be used as biomarkers to distinguish between ECD and LCH patients.

## 4. Materials and Methods

### 4.1. Samples

Plasma samples were collected from 15 HC, 32 untreated ECD patients, and 7 untreated LCH patients at Assuta Medical Center (Tel-Aviv, Israel), Memorial Sloan Kettering Cancer Center (MSK, New York, NY, USA), and Pitié-Salpêtrière Hospital (Paris, France) in accordance with the local Institutional Review Board protocols. Informed consent was obtained from all subjects. Blood samples were centrifuged at 1900× *g* for 15 min and kept at −80 °C.

Excised lesions were fixed in 4% neutral-buffered formalin, embedded in paraffin, and processed by the routine procedures at each local department of pathology.

### 4.2. Genomic Analyses

Genomic analyses were performed on DNA extracted from histiocyte tissue biopsies, as previously describe by Durham et al. [94].

### 4.3. MiRNA Purification

MiRNAs were purified from human plasma and formalin fixed paraffin embedded (FFPE) tissue samples using the miRNeasy Serum/Plasma kit and miRNeasy FFPE kit (Qiagen, Hilden, Germany), respectively, according to the manufacturer’s protocol. RNA quantity was assessed using the Qubit spectrophotometer (Thermo Fisher Scientific Inc., Waltham, MA, USA).

### 4.4. NanoString Analysis

The multiplexed nCounter^®^ miRNA Expression Assay kit (NanoString Technologies, Seattle, WA, USA) was used to profile 800 known mature human miRNAs. Overall, 100 ng of RNA was used as input material with 3 µL of 3-fold diluted sample. A specific DNA tag was ligated onto the 3′ end of each mature miRNA in order to exclusively identify each of the miRNA species present in the sample. Tagging was performed in a multiplexed ligation reaction utilizing reverse complementary bridge oligonucleotides. All hybridization reaction mixtures were incubated at 64 °C for 18 h. Then, excess tags were removed, and the resulting material was hybridized with a panel of fluorescently labeled, barcoded reporter probes that were specific to the miRNA of interest. MiRNA abundance was quantified with the nCounter^®^ Prep Station by counting individual fluorescent barcodes and identifying the target miRNA molecules present in each sample. Each sample was normalized to the geometric mean of the top 100 highest expressed miRNAs. The mean value of the negative controls was set as the lower threshold for each sample; thus, when ≥50% of the value was equal to, or lower than the lower threshold, the miRNA was excluded. Following data preprocessing and normalization, differential expression analysis was performed using the Bioconductor R (DEseq2), as described by Pillar et al. [95].

### 4.5. Quantitative Real-Time PCR (qRT-PCR) Validation

Detection of miRNAs was performed using the TaqMan^®^ Small-RNA primer and probe sets (Applied Biosystems, Foster City, CA, USA, Thermo Fisher Scientific Inc.), as previously describe [96]. qRT-PCR was performed in duplicate by Step One Plus Real-Time PCR (Life Technologies, Thermo Fisher Scientific Inc.) under the following conditions: 95 °C for 20 s, followed by 40 cycles of 95 °C for 1 s, and 60 °C for 20 s. Each value of miRNA expression was represented relative to the expression of external synthetic cel-miR-39 (Applied Biosystems, Thermo Fisher Scientific Inc.), which was used as an internal control. The fold change was calculated using the ΔΔCt method (Applied Biosystems™ Analysis Software, Relative Quantification Analysis Module, v4.1, Applied Biosystems, Foster City, CA, USA, Thermo Fisher Scientific Inc.).

### 4.6. Statistical Analysis

Differential expression analysis of experimental groups was analyzed by a two-tailed Mann–Whitney rank sum test with the Benjamini–Hochberg false discovery rate (FDR) multiple testing correction method. Differences in miRNA expression were statistically evaluated by Student’s *t*-test.

## 5. Conclusions

Our findings indicate that decreased miRNA expression in ECD may lead to upregulation of target genes that participate in inflammation and cell survival signaling. This differential miRNA expression in ECD as compared with healthy controls suggests that there is an additional layer of regulation, that to the best of our knowledge, has never been described before in ECD. This aberrant regulation may lead to upregulation of target key proteins, which may be involved in histiocytosis neoplasms.

## Figures and Tables

**Table 1:** ECD and LCH patient characteristics (Plasma samples).

Name	Gender	Age (Years)	Involved Sites	Kinase Alteration
ECD1	M	75	Bone, Peri-aortic Soft Tissue, Retroperitoneum	BRAF V600E
ECD2	M	35	Bone, Brain, Cavernous sinus, Peri-aortic Soft Tissue, Retroperitoneum, Right Atrium, Skull Base	BRAF V600E
ECD3	M	50	Bone, Brain, Peri-aortic Soft Tissue, Retroperitoneum, Right atrium	BRAF V600E
ECD4	M	75	Bone, Orbit, Retroperitoneum	BRAF V600E
ECD5	M	58	Bone, Orbit, Peri-aortic Soft Tissue, Retroperitoneum	BRAF V600E
ECD6	M	55	Bone, Brain, Heart, Retroperitoneum, Testes	BRAF V600E
ECD7	F	56	Bone, Brain, Retroperitoneum, Orbit	BRAF V600E
ECD8	F	83	Bone, Dura, Orbit, Retroperitoneum, Skin	BRAF V600E
ECD9	M	53	Bone, Peri-aortic, Retroperitoneum	BRAF V600E
ECD10	F	52	Bone, Brain	BRAF V600E
ECD11	M	38	Bone, Brain, Dura, Heart, Peri-aortic, Retroperitoneum, Skin, Skull base	BRAF V600E
ECD12	M	46	Bone, Brain, Retroperitoneum	BRAF V600E
ECD13	M	67	Bone, Dura, Lymph Nodes, Peri-aortic, Retroperitoneum	NRAS Q61R
ECD14	F	66	Bone, Heart	KRAS G12S
ECD15	M	69	Bone, Retroperitoneum, Subcutaneous Soft Tissues	MAP2K2 Y134H
ECD16	M	69	Bone, Brain, Peri-aortic, Retroperitoneum	MAP2K1 C121S
ECD17	M	18	Bone, Brain, Dura	BRAF V600E
ECD18	F	77	Bone, Brain	BRAF V600E
ECD19	M	51	Bone, Larynx, Subcutaneous Soft Tissues	KRAS G12R/ARAF P216A
ECD20	M	57	Bone, Pituitary, Retroperitoneum, Spleen	MAP2K1 Q56P
ECD21	M	54	Bone, Brain, Heart, Retroperitoneum	BRAF V600E
ECD22	M	47	Bone, Brain, Heart, Pleura, Retroperitoneum, Spine	KRAS R149G
ECD23	F	59	Bone, Heart, Lymph Nodes, Pleura, Retroperitoneum, Skin	Unknown
ECD24	F	66	Bone, Heart, Peri-aortic	MAP2K1 P124Q
ECD25	F	35	Bone, Brain	BRAF N486_P490del
ECD26	M	48	Bone, Mucosa	Unknown
ECD27	F	45	Adrenal, Bone, Heart	BRAF V600E
ECD28	M	58	Brain, Sinus, Skin	BRAF V600E
ECD29	M	63	Kidney, Skin	Unknown
ECD30	M	76	Bone, Brain, Sinus	BRAF V600E
ECD31	F	39	Heart	BRAF V600E
ECD32 *	F	76	Brain, Heart, Kidney, Lungs	BRAF V600E
LCH1	F	40	Bone, Lung	BRAF V600E
LCH2	F	72	Bone, Brain, Skin, Vulva	BRAF V600E
LCH3	F	34	Bone	BRAF V600E
LCH4	F	34	Bone, Lymph Nodes, Skin, Subcutaneous Soft Tissues	BRAF V600E
LCH5	M	57	Colon, Oral Mucosa, Skin	BRAF N486_P490del
LCH6	M	58	Bone, Lymph Node, Mastoid, Pancreas, Spine, Submandibular gland	BICD2-BRAF fusion
LCH7	M	33	Bone, Lung, Skin	BRAF V600E

ECD, Erdheim-Chester Disease; M, Male; F, Female; LCH, Langerhans Cell Histiocytosis; * Patient ECD32 has ECD + Chronic myelomonocytic leukemia (CMML).

**Table 2:** ECD and LCH patient characteristics (FFPE samples from lesion site).

Name	Gender	Age (Years)	Involved Sites	Kinase Alteration	Biopsy Site
ECD15	M	69	Bone, retroperitoneum, subcutaneous soft tissues	MAP2K2 Y134H	Tibia
ECD21	M	54	Bone, brain, heart, retroperitoneum	BRAF V600E	Cerebellum
ECD33 *	M	34	Bone, brain, gallbladder and bile ducts, Kidney, lungs, omentum, mesenterium retroperitoneum	BRAF V600E	Omentum
ECD34	M	75	Kidney, pancreas, peri-aortic, retroperitoneum	Unknown	Peri-nepheric
ECD35	M	67	Aorta, kidney	MAP2K1 C121S	Peri-nepheric
LCH8 *	M	34	Bone, brain, gallbladder and bile ducts, kidney, lungs, omentum retroperitoneum	BRAF V600E	Gall bladder
LCH9	F	53	Rib	BRAF V600E	Rib
LCH10	F	24	Gums, lungs	BRAF V600E+ FAT1	Oral Mucosa
LCH11	F	30	Bone, mucosa, scalp	MAP2K1 E102_I103 del	Gingiva

ECD, Erdheim-Chester Disease; M, Male; F, Female; LCH, Langerhans Cell Histiocytosis; * Samples ECD33 and LCH8 are derived from the same patient (mixed ECD/LCH). ECD33 represent the lesion from the ECD compartment and LCH8 represent the lesion from the LCH compartment.

## Supplementary Materials

The following are available online at https://www.mdpi.com/2072-6694/12/11/3240/s1, Table S1: Down regulated miRNAs in ECD patients compare to healthy controls (Nanostring analysis); Table S2: Up regulated miRNAs in ECD patients compare to healthy controls (Nanostring analysis); Table S3: The KEGG pathways significantly enriched for target genes of the top deregulated miRNAs; Figure S1: Full list of enriched Kegg signaling pathways for the downregulated miRNAs represented in Figure 2 ordered by their *p*-values (low to high). Signaling pathway were identified by the web-based computational tool DIANA-miRPath [50]; Figure S2: Validation of miRNAs by qRT-PCR. MiRNA expression in ECD and LCH patients’ plasma samples compared to plasma samples from healthy control (HC). MiRNA expression was normalized to spike-in control cel-miR-39. * *p* < 0.05. Due to the lack of biological material miR-630 was evaluated in 3 LCH plasma samples and not 7 plasma samples. RQ; relative quantification; Figure S3: MiRNA expression before and after treatment with MAPK pathways inhibitors (cobimetinib and vemurafenib). The graph shows the up regulation of let-7a, let-7b, let-7d and let-7g after treatment with MEK inhibitor for 16 weeks. MiRNA expression was measured by qRT-PCR, normalized to spike-in control cel-miR-39. Fold change is shown on the right side of the line. RQ; relative quantification.
