# Supplementary material for: The Contribution of MicroRNAs to the Inflammatory and Neoplastic Characteristics of Erdheim–Chester Disease
Source: Cancers (Basel). 2020 Nov 3;12(11):3240. doi: 10.3390/cancers12113240 (PMC7693724; doi:10.3390/cancers12113240)
Supplement: Supplementary file 1 [file cancers-12-03240-s001.pdf]

cancers

Supplementary Materials: The Contribution of MicroRNAs to the Inflammatory and Neoplastic Characteristics of Erdheim–Chester Disease

Ran Weissman, Eli L. Diamond, Julien Haroche, Nir Pillar, Guy Shapira, Benjamin H. Durham, Justin Buthorn, Fleur Cohen, Michelle Ki, Galia Stemer, Gary A. Ulaner, Zahir Amoura, Jean-François Emile, Roei D. Mazor, Noam Shomron, Omar I. Abdel-Wahab, Ofer Shpilberg and Oshrat Hershkovitz-Rokah

Table S1. Down regulated miRNAs in ECD patients compare to healthy controls (Nanostring analysis).

miRNA ID	Log2 Fold change	P. adjust	miRNA ID	Log2 Fold change	P. adjust
let-7a-5p	-13.3339	1.41E-36	miR-361-5p	-4.87096	1.21E-05
miR-15a-5p	-9.0323	4.92E-24	miR-93-5p	-4.76397	1.40E-05
let-7i-5p	-8.06062	1.21E-21	miR-125a-5p	-3.90587	2.75E-05
miR-107	-8.4171	9.95E-20	miR-106b-5p	-4.82895	2.87E-05
let-7d-5p	-8.2349	7.43E-19	miR-127-3p	-4.00324	3.13E-05
let-7f-5p	-9.74247	3.67E-18	miR-374b-5p	-5.09678	4.31E-05
miR-126-3p	-7.2534	3.01E-16	miR-324-5p	-4.15821	4.31E-05
miR-98-5p	-8.2415	9.23E-16	miR-151a-5p	-5.48352	4.78E-05
let-7g-5p	-7.60983	1.09E-15	miR-652-3p	-5.16788	4.83E-05
let-7e-5p	-7.99647	1.97E-15	miR-543	-3.56585	5.54E-05
miR-221-3p	-6.99156	6.56E-14	miR-26b-5p	-3.35131	6.87E-05
miR-106a-5p	-7.01788	1.69E-13	miR-151a-3p	-4.18411	8.18E-05
miR-17-5p	-7.01788	1.69E-13	miR-148b-3p	-2.60648	0.0001165
miR-18a-5p	-6.87523	1.21E-12	miR-146a-5p	-4.33116	0.0001248
let-7b-5p	-4.27954	3.15E-12	miR-122-5p	-5.07119	0.0001285
miR-142-3p	-6.26377	3.78E-12	miR-335-5p	-4.99793	0.0001687
miR-24-3p	-7.5814	6.96E-12	miR-423-3p	-4.07721	0.0001827
miR-432-5p	-6.85837	9.19E-12	miR-331-3p	-5.56803	0.0002021
miR-26a-5p	-5.63979	9.83E-12	miR-181a-5p	-4.31581	0.0002207
miR-199a-3p	-5.63979	9.83E-12	miR-486-3p	-5.23185	0.0002437
miR-199b-3p	-5.31456	9.83E-12	miR-30c-5p	-3.66999	0.0002437
miR-374a-5p	-6.70424	3.11E-11	miR-382-5p	-4.66976	0.0002495
let-7c-5p	-5.55456	2.11E-10	miR-485-3p	-4.46358	0.0002981
miR-99b-5p	-6.38135	2.55E-10	miR-421	-3.62368	0.0003856
miR-340-5p	-5.55811	6.39E-09	miR-483-3p	-6.14583	0.0006613
miR-28-5p	-5.58429	8.42E-09	miR-19b-3p	-3.4514	0.0007164
miR-130a-3p	-5.79106	8.72E-09	miR-379-5p	-5.24581	0.00118105
miR-23b-3p	-5.80214	9.84E-09	miR-744-5p	-1.86176	0.00118105
miR-20a-5p	-5.67497	1.87E-08	miR-664a-3p	-2.60459	0.00119033
miR-20b-5p	-5.67497	1.87E-08	miR-148a-3p	-3.05061	0.00167956
miR-27b-3p	-6.31287	3.23E-08	miR-503-5p	-5.29262	0.00184066
miR-30b-5p	-6.43225	4.89E-08	miR-593-3p	-5.51412	0.0022199
miR-191-5p	-5.39523	1.05E-07	miR-484	-4.34816	0.00253321
miR-139-3p	-4.84679	1.39E-07	miR-342-3p	-3.09982	0.00325882

**miR-16-5p**	-5.3219	1.60E-07	**miR-125b-5p**	-4.10364	0.00710726
**miR-301a-3p**	-5.64575	1.75E-07	**miR-223-3p**	-2.28696	0.00786213
**miR-185-5p**	-5.34676	5.12E-07	**miR-219a-2-3p**	-4.61708	0.00832234
**miR-766-3p**	-5.94129	7.18E-07	**miR-28-3p**	-3.21419	0.01081694
**miR-144-3p**	-5.64974	1.02E-06	**miR-140-5p**	-3.30094	0.01136544
**miR-1260b**	-6.18322	1.07E-06	**miR-29a-3p**	-2.60509	0.01372143
**miR-5196-3p**	-7.41814	4.85E-06	**miR-92a-3p**	-2.7955	0.01428555
**miR-6732-3p**	-7.41814	4.85E-06	**miR-376a-3p**	-1.55376	0.01479919
**miR-197-3p**	-5.81178	5.20E-06	**miR-22-3p**	-2.58052	0.02044717
**miR-15b-5p**	-4.46294	5.66E-06	**miR-25-3p**	-2.57772	0.02431916
**miR-21-5p**	-4.66871	9.25E-06	**miR-30e-3p**	-3.42976	0.02815174
**miR-199a-5p**	-5.60902	9.41E-06	**miR-363-3p**	-2.49455	0.03154925
**miR-19a-3p**	-4.01594	9.41E-06	**miR-6721-5p**	-3.49232	0.03448361
**miR-23a-3p**	-3.47057	1.11E-05	**miR-625-5p**	-3.45757	0.04456632

Table S2. Up regulated miRNAs in ECD patients compare to healthy controls (Nanostring analysis).

miRNA ID	Log2 Fold change	P. adjust	miRNA ID	Log2 Fold change	P. adjust
**miR-320e**	5.5023	9.20E-18	**miR-630**	6.280959	0.0021169
**miR-514b-5p**	2.805325	0.0011903	**miR-4286**	2.363063	0.0092955
**miR-765**	6.084912	0.0013310			

Table S3. The KEGG pathways significantly enriched for target genes of the top deregulated miRNAs.

Pathway	miRNAs involved	Target genes	P value
**Signaling pathways regulating pluripotency of stem cells**	miR-26a-5p, miR-144-3p, miR-374b-5p, miR-24-3p, miR-374a-5p, miR-340-5p, miR-28-5p, miR-185-5p, miR-107, miR-199a-3p, miR-23b-3p, miR-23a-3p, miR-125a-5p, miR-199a-5p, miR-15a-5p, miR-27b-3p, miR-16-5p, miR-15b-5p, let-7a-5p, let-7i-5p, let-7f-5p, miR-98-5p, let-7g-5p, miR-106a-5p, let-7b-5p, let-7c-5p, miR-20a-5p, miR-361-5p, miR-106b-5p, miR-130a-3p, miR-30b-5p, miR-301a-3p, miR-19a-3p, miR-197-3p, let-7d-5p, let-7e-5p, miR-766-3p, miR-432-5p, miR-21-5p, miR-18a-5p, miR-1260b, miR-93-5p, miR-142-3p, miR-221-3p, miR-191-5p	BMI1, FZD7, PCGF6, JARID2, GSK3B, WNT16, STAT3, DVL3, FZD5, OTX1, ID2, WNT7A, KAT6A, PAX6, INHBC, SMAD2, NRAS, INHBB, SMAD9, APC, HOXB1, PIK3CB, WNT10B, REST, ACVR1B, WNT5A, BMPR1B, PIK3R5, MAPK14, TBX3, HAND1, FZD6, RAF1, SMAD3, SMARCAD1, WNT2B, INHBA, WNT4, WNT3, IGF1R, ZFHX3, ID4, WNT5B, KRAS, FZD3, ACVR1, ACVR2B, PCGF5, FZD4, RIF1, PIK3CD, PIK3R3, FZD10, JAK2, SMAD4, DVL1, AXIN2, AKT1, MYC, LIFR, SKIL, ZIC3, SMAD5, PIK3R1, JAK3, ACVR2A, FGF2, BMP2, WNT11, ACVR1C, IGF1, MAPK12, AKT3, BMPR1A, WNT3A, PIK3CA, WNT8B, IL6ST, MYF5, MAP2K1, ISL1, ID3, WNT9B, DUSP9, FGFR2, FGFR1, FGFR1, SOX2, MAPK1, PCGF3, GRB2, SMAD1, WNT7B, DVL2, KLF4, JAK1, WNT9A, BMP4, MEIS1, BMPR2, COMMD3-BMI1	2.14E-07

**Ras signaling pathway**	miR-26a-5p, let-7g-5p, miR-432-5p, miR-199a-3p, miR-27b-3p, miR-185-5p, miR-361-5p, miR-142-3p, miR-340-5p, miR-125a-5p, miR-15a-5p, miR-106a-5p, miR-20a-5p, miR-144-3p, miR-106b-5p, miR-197-3p, miR-93-5p, miR-374a-5p, miR-30b-5p, miR-16-5p, miR-766-3p, miR-15b-5p, miR-374b-5p, let-7a-5p, let-7f-5p, miR-98-5p, let-7e-5p, let-7b-5p, let-7c-5p, miR-23b-3p, miR-19a-3p, miR-23a-3p, miR-21-5p, miR-130a-3p, miR-301a-3p, miR-18a-5p, miR-221-3p, miR-24-3p, let-7i-5p, miR-107, let-7d-5p, miR-199a-5p, miR-99b-5p, miR-324-5p, miR-28-5p, miR-1260b	PRKCA, FGF12, KSR2, PDGFRA, GNG13, NFKB1, MET, LAT, RIN1, FIGF, SOS2, KSR1, FGF14, NRAS, STK4, RASA2, CALM3, CALM1, PIK3CB, PAK2, PIK3R5, RAP1A, ETS2, RASGRF2, ETS1, PAK7, GNG12, ANGPT2, RALA, FGF10, PLA2G4F, GNB3, RAF1, CHUK, FOXO4, PLD1, FGF4, TIAM1, RASGRP2, EFNA3, IGF1R, EGFR, MRAS, EFNA2, FGF20, RHOA, KRAS, PAK3, CALM2, RRAS2, FASLG, PAK1, EFNA4, IKBKB, FGF11, RAB5A, MLLT4, GNG10, PLA2G3, RASA1, MAPK9, GNB2, PIK3CD, PIK3R3, PLA2G12A, RASGRP1, MAPK8, AKT1, PLA2G16, RGL2, REL, PDGFB, BRAP, PRKCG, FLT1, KIT, VEGFC, GNG2, PIK3R1, SOS1, FGF9, PTPN11, PRKX, PLA2G4A, TBK1, EPHA2, KITLG, RAC1, INSR, RASA3, PAK4, FGF2, CDC42, PAK6, FGF18, NF1, NGF, FGF5, PLD2, PRKCB, RASGRF1, IGF1, GAB1, SHC4, BCL2L1, AKT3, PDGFC, PAK6, FGF21, ANGPT1, PDGFD, PIK3CA, RALGDS, SYNGAP1, GAB2, MAP2K1, RASAL2, RASGRP3, RGL1, FGF23, HGF, VEGFB, RAB5C, FGFR2, PLA2G4E, RELA, VEGFA, HTR7, FGFR1, CSF1R, FGF16, MAPK1, FGF1, ABL2, FGF7, KDR, CSF1, GNG5, GRIN2A, GRB2, RAP1B, GNB4, TEK, RAB5B, NGFR, RAPGEF5, ABL1, GNB5, PLA2G4C, PLA2G2C, MAPK10, PDGFRB, PRKACB, PDGFA, GRIN2B	5.05E-07

**MAPK signaling pathway**	miR-15a-5p, miR-199a-3p, miR-374a-5p, miR-340-5p, miR-16-5p, miR-144-3p, miR-15b-5p, miR-361-5p, miR-197-3p, miR-30b-5p, miR-93-5p, let-7a-5p, let-7f-5p, let-7g-5p, miR-125a-5p, miR-23b-3p, miR-23a-3p, miR-106a-5p, miR-26a-5p, miR-19a-3p, miR-374b-5p, miR-20a-5p, miR-27b-3p, miR-106b-5p, miR-221-3p, miR-24-3p, miR-766-3p, miR-130a-3p, miR-301a-3p, miR-199a-5p, miR-99b-5p, miR-21-5p, miR-185-5p, miR-28-5p, let-7i-5p, let-7d-5p, miR-98-5p, let-7e-5p, let-7b-5p, let-7c-5p, miR-107, miR-142-3p, miR-18a-5p, miR-432-5p, miR-324-5p, miR-191-5p	TAOK3, BRAF, FGF12, FOS, NTRK2, PRKCA, NTF3, CACNG8, PDGFRA, TAB1, CACNA1A, TGFBR1, CACNA2D3, NFKB1, CACNA1G, GNA12, IL1R1, SOS2, MAP4K2, ATF2, FLNC, FGF14, NRAS, DUSP2, STK4, RASA2, ELK4, MAP3K3, CRK, MAPK7, PAK2, MAP2K7, CACNB4, HSPA1L, CACNG7, RAP1A, DUSP6, MAPK14, PTPRR, RASGRF2, GNG12, LAMTOR3, FGF10, PPP3R1, PLA2G4F, RAF1, CHUK, MAP4K3, MAP4K4, MAP2K3, RPS6KA1, FGF4, RASGRP2, EGFR, GADD45A, MRAS, FGF20, MAP3K4, MAP3K1, MAP3K13, TAB2, KRAS, BDNF, MAP3K11, RRAS2, FASLG, TAOK1, PAK1, TP53, MAP2K6, IKBKB, FGF11, PPP3CA, NLK, DUSP10, RASA1, MAPK9, CASP3, JUN, PPP3CB, RASGRP1, PPP5D1, RAPGEF2, MAPK8, TRAF6, AKT1, MYC, NTRK1, PPM1A, MAPKAPK3, ZAK, PDGFB, CACNB1, PRKCG, FLNA, CACNA1E, CACNG4, RPS6KA6, SOS1, FGF9, PRKX, PLA2G4A, RAC1, DUSP8, FGF2, CDC42, STK3, CACNA2D4, FGF18, HSPA8, NF1, NGF, FGF5, DUSP7, PRKCB, MAX, FAS, RASGRF1, RPS6KA3, TGFB2, MAPK12, AKT3, FGF21, TNFRSF1A, MECOM, MAP2K1, STMN1, MAP3K2, MKNK1, MAP3K8, RASGRP3, PPM1B, CACNA2D1, CACNB2, MEF2C, FGF23, MKNK2, DUSP5, DUSP9, FGFR2, HSPA1B, IL1A, MAP2K4, RPS6KA4, PLA2G4E, RELA, CACNA1D, NFATC3, FGFR1, DUSP16, FGF16, MAPK1, MAP3K7, FGF1, SRF, NFATC1, FGF7, GRB2, RAP1B, DUSP1, TGFBR2, PLA2G4C, MAP3K5, MAPK10, PDGFRB, ARRB1, PRKACB, PPP3R2, CACNB3, PDGFA, GNA12	5.26E-06
**Hippo signaling pathway**	miR-106a-5p, miR-374a-5p, miR-340-5p, miR-130a-3p, miR-20a-5p, miR-27b-3p, miR-191-5p, miR-301a-3p, miR-21-5p, miR-19a-3p, miR-361-5p, miR-106b-5p, miR-374b-5p, miR-125a-5p, let-7a-5p, miR-15a-5p, let-7i-5p, let-7f-5p, miR-98-5p, let-7g-5p, let-7e-5p, miR-18a-5p, let-7b-5p, let-7c-5p, miR-16-5p, miR-185-5p, miR-15b-5p, miR-93-5p, miR-199a-5p, let-7d-5p, miR-24-3p, miR-432-5p, miR-28-5p, miR-30b-5p, miR-23b-3p, miR-766-3p, miR-23a-3p, miR-142-3p, miR-26a-5p, miR-144-3p, miR-199a-3p, miR-107, miR-221-3p, miR-197-3p	FZD7, ACTB, GSK3B, DVL3, WNT16, FZD5, TGFBR1, YWHAH, ID2, PARD6G, WNT7A, YAP1, SMAD2, YWHAE, BTRC, APC, WNT10B, PPP2CA, WNT5A, DLG1, BMPR1B, YWHAG, PPP1CC, CCND2, TCF7L1, FZD6, SMAD3, WNT2B, WNT4, MOB1B, WNT3, YWHAB, CRB1, TEAD3, PPP2R2B, WWC1, PPP2R2D, WWTR1, WNT5B, TP53BP2, FZD3, BMP8B, YWHAQ, MPP5, LLGL2, FZD4, DLG4, FZD10, CCND1, SMAD4, DVL1, AXIN2, TP73, NKD1, PPP2R2A, MYC, RASSF6, PPP2R1A, FRMD6, SAV1, TEAD1, YWHAZ, GDF6, CRB2, STK3, BMP2, WNT11, TGFB2, BBC3, BMPR1A, CTNNA3, WNT3A, WNT8B, DLG2, LEF1, SMAD7, WNT9B, MOB1A, TCF7, LATS1, SOX2, FGF1, LATS2, FBXW11, PPP2R1B, SMAD1, PARD6B, WNT7B, DVL2, INADL, TGFBR2, PRKCZ, WNT9A, BMP4, BMPR2, CCND3, AJUBA, CTGF, PPP1CB	9.01E-06

**Wnt signaling pathway**	miR-106a-5p, miR-20a-5p, miR-19a-3p, miR-106b-5p, miR-766-3p, miR-26a-5p, miR-27b-3p, miR-340-5p, let-7a-5p, let-7i-5p, let-7d-5p, let-7f-5p, miR-98-5p, let-7g-5p, let-7e-5p, let-7b-5p, miR-125a-5p, miR-24-3p, miR-30b-5p, miR-185-5p, miR-142-3p, miR-93-5p, miR-221-3p, miR-144-3p, miR-197-3p, miR-199a-5p, miR-361-5p, miR-15a-5p, miR-16-5p, miR-130a-3p, miR-301a-3p, miR-107, miR-23b-3p, miR-23a-3p, miR-432-5p, miR-374a-5p, miR-374b-5p, miR-199a-3p, miR-28-5p, miR-21-5p, miR-324-5p, miR-1260b, miR-18a-5p	FZD7, CTNNBIP1, CAMK2D, DAAM2, GSK3B, PRKCA, DVL3, CSNK2A2, WNT16, FZD5, LRP6, TBL1X, WNT7A, BTRC, APC, VANGL1, WNT5A, CTBP1, CHD8, PORCN, CCND2, DKK2, ROCK2, TCF7L1, FZD6, PPP3R1, SMAD3, WNT2B, WNT4, LRP5, RHOA, WNT5B, FZD3, SKP1, TP53, NFATC4, FRAT2, PPP3CA, PRICKLE1, NLK, PLCB1, FZD4, SENP2, MAPK9, GPC4, JUN, FZD10, CCND1, SMAD4, PPP3CB, DVL1, AXIN2, NFATC2, MAPK8, NKD1, CSNK1A1, MYC, VANGL2, PRKCG, CSNK2B, CSNK2A1, PRKX, RAC1, SIAH1, WNT11, PSEN1, PRKCB, PRICKLE2, EP300, CAMK2B, CXXC4, WNT3A, WNT8B, LEF1, BAMBI, SFRP1, WIF1, WNT9B, FOSL1, TCF7, NFATC3, DAAM1, MAP3K7, CREBBP, FBXW11, NFATC1, WNT7B, DVL2, TBL1XR1, WNT9A, PLCB4, PLCB2, CCND3, MAPK10, PRKACB, PPP3R2	1.03E-05
**Glutamatergic synapse**	miR-26a-5p, miR-340-5p, miR-125a-5p, miR-766-3p, miR-324-5p, miR-19a-3p, miR-15a-5p, miR-107, miR-432-5p, miR-16-5p, miR-15b-5p, miR-18a-5p, miR-185-5p, miR-23b-3p, miR-23a-3p, miR-199a-3p, miR-27b-3p, miR-30b-5p, miR-197-3p, miR-28-5p, miR-106a-5p, miR-106b-5p, miR-374b-5p, miR-93-5p, let-7g-5p, miR-361-5p, miR-24-3p, miR-21-5p, miR-374a-5p, miR-142-3p, let-7f-5p, let-7b-5p, let-7c-5p, miR-130a-3p, miR-301a-3p, miR-199a-5p, let-7a-5p, let-7d-5p, miR-98-5p, let-7e-5p, let-7i-5p	SLC38A1, PRKCA, GRIN3A, CACNA1A, GNG13, ADCY1, ADRBK1, ADCY7, ADRBK2, ADCY2, TRPC1, GRM5, SLC17A8, GNG12, PPP3R1, PLA2G4F, GNB3, SLC1A1, PLD1, SLC17A6, GRIA1, GNAI3, GLS, HOMER2, GRM6, GRIK5, GRIA2, PPP3CA, GNG10, PLCB1, DLG4, PPP3CB, DLGAP1, PRKCG, SLC38A2, GNG2, ITPR1, SLC17A7, PRKX, PLA2G4A, GRM7, PLD2, PRKCB, GNAQ, GRIA4, GNAI2, GRIN2D, HOMER1, GRIK4, SLC1A3, SHANK2, GRIK2, GRM4, PLA2G4E, ITPR3, CACNA1D, GRM8, MAPK1, ITPR2, GNG5, GRIN2A, GLS2, ADCY9, ADCY4, GNB4, KCNJ3, PLCB4, PLCB2, GNB5, PLA2G4C, SLC1A2, PRKACB, PPP3R2, GRIA3, ADCY6, GRIK3, GRIN2B	3.27E-05
**Rap1 signaling pathway**	miR-27b-3p, miR-340-5p, miR-23b-3p, miR-23a-3p, miR-374a-5p, miR-144-3p, miR-19a-3p, miR-125a-5p, miR-374b-5p, miR-432-5p, miR-130a-3p, miR-301a-3p, miR-24-3p, miR-221-3p, miR-15a-5p, miR-107, miR-106a-5p, miR-18a-5p, miR-26a-5p, miR-20a-5p, miR-16-5p, miR-15b-5p, miR-106b-5p, miR-197-3p, miR-28-5p, miR-185-5p, miR-766-3p, miR-361-5p, miR-30b-5p, let-7e-5p, miR-21-5p, let-7a-5p, miR-98-5p, miR-199a-3p, miR-99b-5p, miR-93-5p, let-7i-5p, let-7f-5p, let-7g-5p, let-7b-5p, let-7c-5p, miR-142-3p, let-7d-5p, miR-324-5p	BRAF, ACTB, MAGI2, FGF12, PRKCA, PDGFRA, MET, LAT, SIPA1L3, ADCY1, ITGB1, PARD6G, FIGF, CTNND1, FGF14, ADCY7, NRAS, ADORA2B, ADCY2, CALM3, CALM1, LPAR3, CRK, THBS1, PIK3CB, SKAP1, FPR1, PIK3R5, RAP1A, MAPK14, ANGPT2, RALA, FGF10, RAF1, MAGI3, MAP2K3, FGF4, TIAM1, RASGRP2, EFNA3, IGF1R, EGFR, GNAI3, MRAS, EFNA2, FGF20, F2RL3, RHOA, KRAS, SPECC1L-ADORA2A, CALM2, ADORA2A, MAP2K6, LPAR4, EFNA4, SIPA1L1, RAPGEF4, VAV2, FGF11, MLLT4, KRIT1, PLCB1, PIK3CD, PIK3R3, PFN2, LPAR1, RAPGEF2, AKT1, F2R, RAPGEF6, PDGFB, PRKCG, FLT1, KIT, VEGFC, PIK3R1, DOCK4, FGF9, FARP2, CNR1, EPHA2, KITLG, RAC1, INSR, FGF2, CDC42, FGF18, NGF, FGF5, PRKCB, FYB, IGF1, GNAQ, MAPK12, AKT3, PDGFC, MAGI1, FGF21, ANGPT1, PDGFD, GNAI2, PIK3CA, RALGDS, MAP2K1, PRKD3, RASGRP3, SIPA1L2, FGF23, HGF, VEGFB, PRKD2, FGFR2, LCP2, VEGFA, FGFR1, CSF1R, FGF16, MAPK1, TLN1, FGF1,	7.35E-05

		FGF7, KDR, CSF1, GRIN2A, ADCY9, ADCY4, PARD6B, RAP1B, TEK, PRKCZ, NGFR, RAPGEF5, ITGAL, PLCB4, PLCB2, PDGFRB, RGS14, PDGFA, ADCY6, ITGB3, GRIN2B	
**Glioma**	let-7a-5p, miR-15a-5p, let-7i-5p, let-7d-5p, let-7f-5p, miR-98-5p, let-7g-5p, let-7e-5p, let-7b-5p, miR-432-5p, let-7c-5p, miR-340-5p, miR-30b-5p, miR-16-5p, miR-15b-5p, miR-107, miR-26a-5p, miR-27b-3p, miR-185-5p, miR-144-3p, miR-106a-5p, miR-18a-5p, miR-374a-5p, miR-23b-3p, miR-20a-5p, miR-23a-3p, miR-106b-5p, miR-374b-5p, miR-361-5p, miR-19a-3p, miR-130a-3p, miR-301a-3p, miR-766-3p, miR-324-5p, miR-199a-3p, miR-197-3p, miR-142-3p, miR-21-5p, miR-93-5p, miR-125a-5p, miR-199a-5p, miR-24-3p	CAMK2D, BRAF, PRKCA, PDGFRA, E2F1, SOS2, E2F2, NRAS, CALM3, CALM1, PIK3CB, TGFA, PIK3R5, RAF1, IGF1R, EGFR, KRAS, CDK6, CALM2, TP53, PIK3CD, PIK3R3, CCND1, E2F3, AKT1, PDGFB, PRKCG, PIK3R1, RB1, SOS1, PRKCB, IGF1, SHC4, AKT3, CAMK2B, PIK3CA, CDKN1A, MAP2K1, MTOR, PTEN, MAPK1, GRB2, MDM2, PDGFRB, PDGFA	0.000407
**PI3K-Akt signaling pathway**	miR-374a-5p, miR-340-5p, miR-15a-5p, miR-16-5p, miR-15b-5p, miR-106a-5p, miR-20a-5p, miR-125a-5p, miR-106b-5p, let-7a-5p, miR-98-5p, miR-18a-5p, miR-26a-5p, miR-30b-5p, miR-27b-3p, let-7e-5p, miR-130a-3p, miR-301a-3p, miR-199a-5p, let-7i-5p, let-7f-5p, let-7g-5p, let-7b-5p, let-7c-5p, miR-197-3p, miR-107, miR-766-3p, miR-126-3p, miR-19a-3p, miR-199a-3p, miR-144-3p, miR-374b-5p, miR-24-3p, let-7d-5p, miR-361-5p, miR-142-3p, miR-432-5p, miR-185-5p, miR-324-5p, miR-21-5p, miR-28-5p, miR-23b-3p, miR-23a-3p, miR-221-3p, miR-93-5p	PHLPP2, PRLR, FGF12, GSK3B, PRKCA, RBL2, TSC1, PDGFRA, GNG13, NFKB1, PPP2R5E, MET, YWHAH, MYB, ITGB1, FIGF, SOS2, ITGB8, LAMB1, ATF2, ITGA9, FGF14, COL6A5, NRAS, PRKAA2, PPP2R3A, YWHAE, LPAR3, THBS1, ITGA8, PIK3CB, THBS2, PPP2CA, CREB5, SYK, COL4A5, LAMB4, CDC37, COL24A1, PIK3R5, YWHAG, MCL1, CDK2, CCND2, COL27A1, ITGB6, GNG12, ANGPT2, FGF10, GNB3, IL7, ITGA5, RAF1, ITGA3, CHUK, LAMA1, BCL2, CDKN1B, FGF4, PPP2R5D, YWHAB, MTCP1, EFNA3, PPP2R2B, IGF1R, EGFR, EFNA2, FGF20, PPP2R2D, TLR4, PPP2R5C, ITGA1, KRAS, CDK6, COL3A1, LPAR6, FASLG, IL7R, IFNAR2, YWHAQ, RPS6KB2, PPP2R5A, TP53, LPAR4, GHR, EFNA4, G6PC3, CREB1, IKBKB, FGF11, IL4, GNG10, PTK2, BRCA1, ITGAV, THEM4, CRTC2, IL4R, DDIT4, GNB2, PIK3CD, PIK3R3, CCND1, JAK2, EIF4E, LPAR1, CCNE2, COL4A2, AKT1, PPP2R2A, F2R, MYC, RELN, PPP2R1A, PDGFB, COL1A1, FLT1, EIF4B, KIT, VEGFC, GNG2, COL4A3, ITGA2, PIK3R1, COL4A4, SOS1, JAK3, IL2RA, FGF9, YWHAZ, PPP2R3C, EPHA2, KITLG, COL11A2, IRS1, RAC1, INSR, PRKAA1, FGF2, CHRM2, FGF18, NGF, FGF5, COL1A2, LAMC1, IGF1, ITGA7, BCL2L1, AKT3, PDGFC, COL11A1, FGF21, EIF4E2, CREB3L2, ANGPT1, CCNE1, PDGFD, PIK3CA, COL4A6, OSM, FOXO3, FN1, PKN2, CDKN1A, MAP2K1, TNR, EPO, PDPK1, ITGA4, IFNA17, FGF23, HGF, MTOR, VEGFB, FGFR2, ITGA6, RELA, VEGFA, PTEN, FGFR1, SGK3, CSF1R, FGF16, OSMR, MAPK1, FGF1, PPP2R1B, FGF7, KDR, CSF1, GNG5, GRB2, COL5A2, GNB4, TEK, SGK1, PRKCZ, JAK1,	0.000410

		MDM2, NGFR, GNB5, BCL2L11, CCND3, PDGFRB, RPS6KB1, TCL1B, COL4A1, CHRM1, IL6R, PDGFA, ITGB3	
**Melanoma**	miR-15a-5p, miR-16-5p, miR-15b-5p, miR-374a-5p, miR-340-5p, miR-27b-3p, miR-30b-5p, miR-144-3p, let-7a-5p, let-7i-5p, let-7f-5p, miR-98-5p, let-7g-5p, let-7b-5p, let-7c-5p, miR-130a-3p, miR-20a-5p, miR-301a-3p, miR-93-5p, miR-106b-5p, miR-107, miR-432-5p, miR-23b-3p, miR-19a-3p, miR-23a-3p, miR-199a-3p, miR-26a-5p, miR-374b-5p, miR-361-5p, miR-125a-5p, let-7e-5p, miR-18a-5p, miR-185-5p, miR-21-5p, let-7d-5p, miR-106a-5p, miR-142-3p, miR-197-3p, miR-199a-5p, miR-24-3p, miR-766-3p	BRAF, FGF12, PDGFRA, E2F1, MET, FGF14, E2F2, NRAS, PIK3CB, PIK3R5, FGF10, RAF1, FGF4, IGF1R, EGFR, FGF20, KRAS, CDK6, TP53, MITF, FGF11, PIK3CD, PIK3R3, CCND1, E2F3, AKT1, PDGFB, PIK3R1, RB1, FGF9, FGF2, FGF18, FGF5, IGF1, AKT3, PDGFC, FGF21, PDGFD, PIK3CA, CDKN1A, MAP2K1, FGF23, HGF, PTEN, FGFR1, FGF16, MAPK1, FGF1, FGF7, MDM2, PDGFRB, PDGFA	0.000889
**T cell receptor signaling pathway**	miR-15a-5p, miR-107, miR-199a-3p, miR-340-5p, miR-16-5p, miR-144-3p, miR-15b-5p, miR-27b-3p, miR-19a-3p, miR-26a-5p, miR-28-5p, miR-185-5p, miR-24-3p, miR-199a-5p, miR-374b-5p, miR-125a-5p, let-7a-5p, let-7i-5p, let-7d-5p, let-7f-5p, miR-98-5p, let-7g-5p, let-7b-5p, let-7c-5p, miR-130a-3p, miR-23b-3p, miR-30b-5p, miR-301a-3p, miR-23a-3p, miR-142-3p, miR-766-3p, miR-106a-5p, miR-20a-5p, miR-106b-5p, miR-21-5p, miR-374a-5p, miR-93-5p, miR-432-5p, miR-221-3p, let-7e-5p, miR-324-5p, miR-18a-5p, miR-361-5p, miR-197-3p	FOS, GSK3B, PRKCQ, NFKB1, LAT, SOS2, CBL, NRAS, NFKBIB, PIK3CB, PAK2, MAP2K7, TEC, DLG1, PIK3R5, MAPK14, PAK7, PPP3R1, CD40LG, RAF1, CHUK, BCL10, RHOA, KRAS, PAK3, FYN, PAK1, VAV2, IKBKB, NCK1, IL4, PPP3CA, CBLB, CD28, MAPK9, PIK3CD, JUN, PIK3R3, NFKBIE, PPP3CB, RASGRP1, NCK2, NFATC2, AKT1, ICOS, PIK3R1, SOS1, PAK4, CDC42, PAK6, MAPK12, AKT3, PAK6, CD4, PIK3CA, CD3G, MALT1, IFNG, MAP2K1, MAP3K8, PDPK1, CTLA4, RELA, LCP2, VAV3, NFATC3, MAPK1, MAP3K7, NFATC1, IL10, GRB2, PDCD1, PPP3R2	0.000978
**mTOR signaling pathway**	miR-30b-5p, let-7a-5p, let-7i-5p, let-7d-5p, let-7f-5p, miR-98-5p, let-7g-5p, let-7b-5p, let-7c-5p, miR-130a-3p, miR-27b-3p, miR-301a-3p, miR-19a-3p, miR-432-5p, miR-23b-3p, miR-23a-3p, miR-24-3p, miR-374a-5p, miR-340-5p, miR-15a-5p, miR-106a-5p, miR-26a-5p, miR-20a-5p, miR-16-5p, miR-144-3p, miR-15b-5p, miR-106b-5p, miR-374b-5p, miR-107, miR-185-5p, miR-18a-5p, miR-199a-3p, miR-125a-5p, miR-766-3p, let-7e-5p, miR-142-3p, miR-361-5p, miR-93-5p, miR-126-3p, miR-28-5p, miR-21-5p, miR-324-5p	BRAF, PRKCA, TSC1, RRAGD, PRKAA2, PIK3CB, PIK3R5, RPS6KA1, RPS6KB2, RICTOR, IKBKB, DDIT4, PIK3CD, PIK3R3, EIF4E, HIF1A, AKT1, RRAGA, PRKCG, EIF4B, RPS6KA6, PIK3R1, IRS1, PRKAA1, ULK3, PRKCB, RPS6KA3, IGF1, AKT3, EIF4E2, PIK3CA, PDPK1, MTOR, ULK1, VEGFA, PTEN, CAB39, MAPK1, RRAGC, ULK2, RRAGB, STRADA, CAB39L, RPS6KB1, RPS6KA2	0.001425

**Focal adhesion**	miR-15a-5p, miR-107, miR-106a-5p, miR-374a-5p, miR-340-5p, miR-23b-3p, miR-27b-3p, miR-16-5p, miR-144-3p, miR-15b-5p, miR-23a-3p, miR-374b-5p, miR-24-3p, miR-20a-5p, miR-19a-3p, miR-106b-5p, let-7a-5p, let-7i-5p, let-7d-5p, let-7f-5p, miR-98-5p, let-7g-5p, let-7e-5p, let-7b-5p, miR-199a-3p, let-7c-5p, miR-30b-5p, miR-361-5p, miR-93-5p, miR-18a-5p, miR-26a-5p, miR-185-5p, miR-432-5p, miR-142-3p, miR-221-3p, miR-197-3p, miR-766-3p, miR-130a-3p, miR-301a-3p, miR-125a-5p, miR-28-5p, miR-199a-5p, miR-324-5p, miR-21-5p, miR-126-3p	BRAF, ACTB, GSK3B, PRKCA, PDGFRA, ACTN2, CAPN2, MYLK4, MET, ITGB1, FIGF, ROCK1, SOS2, ITGB8, LAMB1, FLNC, ITGA9, COL6A5, CRK, THBS1, ITGA8, PIK3CB, PAK2, THBS2, COL4A5, LAMB4, COL24A1, PIK3R5, PPP1CC, RAP1A, CCND2, COL27A1, PXN, ROCK2, ITGB6, PAK7, ITGA5, RAF1, ITGA3, LAMA1, BCL2, IGF1R, EGFR, ZYX, VCL, PPP1R12B, CAV2, ITGA1, PAK3, COL3A1, FYN, PAK1, VAV2, PTK2, ITGAV, PPP1R12A, MAPK9, PIK3CD, JUN, PIK3R3, CCND1, COL4A2, MAPK8, AKT1, PARVA, DIAPH1, RELN, PDGFB, COL1A1, ACTN1, PRKCG, FLT1, VEGFC, FLNA, COL4A3, ITGA2, PIK3R1, COL4A4, SOS1, COL11A2, RAC1, PAK4, CDC42, PAK6, COL1A2, PRKCB, RASGRF1, LAMC1, IGF1, ITGA7, SHC4, AKT3, PDGFC, MYLK3, COL11A1, PAK6, PDGFD, PIK3CA, COL4A6, FN1, MAP2K1, BIRC3, TNR, PDPK1, ITGA4, HGF, VEGFB, ITGA6, VAV3, VEGFA, PTEN, MAPK1, TLN1, KDR, GRB2, RAP1B, COL5A2, ARHGAP5, MYLK, CCND3, ILK, MAPK10, PDGFRB, XIAP, COL4A1, PPP1CB, PDGFA, ITGB3	0.004624
**cAMP signaling pathway**	miR-106a-5p, miR-340-5p, miR-20a-5p, miR-185-5p, miR-27b-3p, miR-374b-5p, miR-26a-5p, miR-23b-3p, miR-23a-3p, miR-361-5p, miR-125a-5p, miR-21-5p, miR-19a-3p, miR-144-3p, miR-106b-5p, miR-130a-3p, miR-301a-3p, miR-374a-5p, miR-142-3p, miR-221-3p, miR-30b-5p, miR-18a-5p, let-7d-5p, miR-199a-3p, miR-93-5p, miR-15a-5p, miR-16-5p, miR-15b-5p, miR-24-3p, miR-766-3p, miR-107, miR-197-3p, miR-432-5p, miR-28-5p, miR-199a-5p, let-7a-5p, let-7i-5p, let-7f-5p, miR-98-5p, let-7g-5p, let-7e-5p, let-7b-5p, let-7c-5p, miR-127-3p	SSTR1, CAMK2D, BRAF, FOS, GRIN3A, PDE4B, NFKB1, ATP1B2, ADCY1, ROCK1, CAMK4, ADCY7, ATP2B2, ADCY2, CNGA4, CALM3, CALM1, PIK3CB, PTCH1, MC2R, CREB5, ATP1A2, HHIP, PIK3R5, PPP1CC, RAP1A, HTR1E, ACOX3, ROCK2, PDE3A, DRD1, RAF1, HTR1F, PLD1, SUCNR1, TIAM1, GRIA1, ATP1B1, ADRB2, CFTR, GNAI3, RHOA, SPECC1L-ADORA2A, BDNF, CALM2, RRAS2, ABCC4, NPY1R, PTGER3, ATP2B1, ADORA2A, PAK1, PDE4D, PDE3B, RAPGEF4, VAV2, CREB1, GRIA2, GABBR2, MLLT4, CNGA3, SLC9A1, PPP1R12A, MAPK9, PIK3CD, JUN, PIK3R3, PPARA, GIPR, CNGB3, SOX9, ATP1A3, RYR2, PLN, MAPK8, AKT1, F2R, PTGER2, TSHR, ATP1A4, PIK3R1, PRKX, RAC1, ATP1B4, CHRM2, FSHB, PLD2, HTR4, EP300, AKT3, CAMK2B, CREB3L2, HCAR1, GRIA4, GNAI2, PIK3CA, GLP1R, GRIN2D, MAP2K1, ATP2B4, EDNRA, ATP2A2, ATP2B3, ATP1A1, RELA, VAV3, CACNA1D, MAPK1, CREBBP, NFATC1, GRIN2A, ADCY9, ADCY4, RAP1B, ADRB1, HCN4, MAPK10, PDE4A, PRKACB, PPP1CB, GRIA3, CHRM1, ADCY6, GRIN2B	0.006027

**Chronic myeloid leukemia**	miR-374b-5p, miR-106a-5p, miR-130a-3p, miR-23b-3p, miR-20a-5p, miR-301a-3p, miR-21-5p, miR-19a-3p, miR-23a-3p, miR-106b-5p, miR-340-5p, miR-27b-3p, miR-30b-5p, miR-15a-5p, miR-107, miR-16-5p, miR-15b-5p, miR-199a-5p, let-7a-5p, let-7i-5p, let-7d-5p, let-7f-5p, miR-98-5p, let-7g-5p, let-7e-5p, let-7b-5p, let-7c-5p, miR-24-3p, miR-125a-5p, miR-432-5p, miR-374a-5p, miR-144-3p, miR-197-3p, miR-142-3p, miR-766-3p, miR-18a-5p, miR-26a-5p, miR-93-5p, miR-199a-3p, miR-185-5p, miR-221-3p	BRAF, E2F1, TGFBR1, NFKB1, SOS2, CBL, E2F2, NRAS, CRK, RUNX1, PIK3CB, CTBP1, PIK3R5, BCR, RAF1, SMAD3, CHUK, CDKN1B, KRAS, CDK6, TP53, IKBKB, CBLB, PIK3CD, PIK3R3, CCND1, SMAD4, E2F3, AKT1, MYC, PIK3R1, RB1, SOS1, PTPN11, TGFB2, SHC4, BCL2L1, AKT3, PIK3CA, GAB2, MECOM, CDKN1A, MAP2K1, RELA, MAPK1, GRB2, TGFBR2, MDM2, ABL1	0.009193
**AMPK signaling pathway**	miR-106a-5p, miR-27b-3p, miR-324-5p, miR-340-5p, miR-28-5p, miR-125a-5p, miR-374a-5p, miR-20a-5p, miR-361-5p, miR-106b-5p, let-7a-5p, miR-15a-5p, let-7i-5p, let-7f-5p, miR-98-5p, let-7g-5p, let-7e-5p, let-7b-5p, let-7c-5p, miR-130a-3p, miR-23b-3p, miR-30b-5p, miR-16-5p, miR-301a-3p, miR-15b-5p, miR-23a-3p, miR-107, miR-199a-3p, miR-144-3p, miR-24-3p, miR-26a-5p, miR-19a-3p, miR-199a-5p, miR-142-3p, miR-766-3p, miR-21-5p, miR-18a-5p, miR-374b-5p, let-7d-5p, miR-432-5p, miR-126-3p, miR-185-5p, miR-1260bmiR-93-5p, miR-221-3p	IRS2, FASN, RAB8A, TSC1, EEF2K, PFKP, PPP2R5E, SLC2A4, SCD5, PRKAA2, PPP2R3A, CCNA2, HMGCR, SIRT1, PIK3CB, PPP2CA, CREB5, PIK3R5, RAB2A, PPP2R5D, CFTR, PPP2R2B, IGF1R, PPP2R2D, PPP2R5C, ADIPOR2, CAMKK2, RPS6KB2, CCNA1, PPP2R5A, G6PC3, CREB1, CRTC2, PIK3CD, PIK3R3, CCND1, TBC1D1, AKT1, PPP2R2A, PRKAG1, MLYCD, PPP2R1A, PIK3R1, PPP2R3C, IRS1, INSR, PRKAA1, ELAVL1, STRADB, PRKAB2, IGF1, PPARG, AKT3, CREB3L2, PPARGC1A, ACACB, PRKAG2, PIK3CA, PFKM, SCD, FOXO3, PDPK1, PFKFB2, LEPR, MTOR, ULK1, FOXO1, CAB39, MAP3K7, PPP2R1B, RAB10, PFKFB4, RAB14, STRADA, CAB39L, PRKAG3, RPS6KB1, PFKFB3	0.021106
**ErbB signaling pathway**	miR-340-5p, let-7a-5p, let-7i-5p, let-7d-5p, let-7f-5p, miR-98-5p, let-7g-5p, let-7e-5p, let-7b-5p, let-7c-5p, miR-107, miR-199a-3p, miR-23b-3p, miR-144-3p, miR-23a-3p, miR-125a-5p, miR-27b-3 p, miR-221-3p, miR-374a-5p, miR-130a-3p, miR-301a-3p, miR-374b-5p, miR-106a-5p, miR-432-5p, miR-26a-5p, miR-20a-5p, miR-19a-3p, miR-361-5p, miR-106b-5p, miR-30b-5p, miR-15a-5p, miR-16-5p, miR-15b-5p, miR-24-3p, miR-142-3p, miR-766-3p, miR-324-5p, miR-185-5p, miR-197-3p, miR-199a-5p, miR-21-5p, miR-93-5p, miR-28-5p, miR-18a-5p	CAMK2D, BRAF, GSK3B, HBEGF, PRKCA, SOS2, CBL, NRAS, CRK, PIK3CB, PAK2, MAP2K7, TGFA, PIK3R5, PAK7, RAF1, CDKN1B, EGFR, KRAS, PAK3, RPS6KB2, PAK1, NCK1, PTK2, CBLB, MAPK9, PIK3CD, JUN, PIK3R3, NCK2, MAPK8, AKT1, MYC, NRG3, PRKCG, PIK3R1, SOS1, PAK4, PAK6, BTC, NRG1, PRKCB, GAB1, SHC4, AKT3, CAMK2B, PAK6, PIK3CA, CDKN1A, MAP2K1, MTOR, MAP2K4, MAPK1, ABL2, GRB2, ABL1, ERBB4, MAPK10, RPS6KB1, EREG	0.022513

**cGMP-PKG signaling pathway**	miR-340-5p, miR-19a-3p, miR-23b-3p, miR-27b-3p, miR-30b-5p, miR-23a-3p, miR-221-3p, miR-144-3p, miR-93-5p, miR-106b-5p, miR-130a-3p, miR-301a-3p, miR-197-3p, miR-374a-5p, miR-18a-5p, miR-374b-5p, miR-142-3p, miR-361-5p, let-7a-5p, let-7i-5p, let-7d-5p, let-7f-5p, miR-98-5p, let-7g-5p, let-7e-5p, let-7b-5p, miR-199a-3p, let-7c-5p, miR-26a-5p, miR-21-5p, miR-126-3p, miR-15a-5p, miR-107, miR-16-5p, miR-15b-5p, miR-766-3p, miR-106a-5p, miR-432-5p, miR-20a-5p, miR-185-5p, miR-24-3p, miR-125a-5p, miR-28-5p, miR-324-5p, miR-127-3p	IRS2, GUCY1B3, MYLK4, ATP1B2, ADCY1, VDAC1, GNA12, MYH7, ROCK1, ATF2, ADCY7, ATP2B2, ADCY2, CALM3, CALM1, KCNJ8, GUCY1A3, PIK3CB, CREB5, ATP1A2, PIK3R5, PPP1CC, GNA13, ROCK2, BDKRB2, PDE3A, PPP3R1, RAF1, MEF2D, ATP1B1, ADRB2, SLC8A2, GNAI3, ADRB3, RHOA, CALM2, PRKCE, ADRA2A, KCNMB1, ATP2B1, NFATC4, SLC8A3, PDE3B, CREB1, PPP3CA, PLCB1, PRKG2, PPP1R12A, PIK3CD, PIK3R3, PPP3CB, ATP1A3, PLN, NFATC2, AKT1, EDNRB, SLC8A1, ATP1A4, ITPR1, KCNMB2, PIK3R1, IRS1, INSR, ATP1B4, SLC25A6, KCNMB4, GNAQ, AKT3, MYLK3, CREB3L2, GUCY1A2, GNAI2, PIK3CA, PDE5A, MAP2K1, ATP2B4, EDNRA, ATP2A2, MEF2C, PPIF, PRKG1, ATP2B3, ATP1A1, ITPR3, CACNA1D, NFATC3, MAPK1, ITPR2, SRF, NFATC1, ADCY9, ADCY4, ADRA1D, MEF2A, ADRB1, PLCB4, PLCB2, MYLK, PPP3R2, PPP1CB, VDAC3, ADCY6	0.024420
**Acute myeloid leukemia**	miR-24-3p, miR-26a-5p, miR-185-5p, miR-144-3p, miR-766-3p, miR-221-3p, miR-340-5p, miR-30b-5p, miR-27b-3p, miR-199a-3p, let-7a-5p, let-7i-5p, let-7d-5p, let-7f-5p, miR-98-5p, let-7g-5p, let-7e-5p, let-7b-5p, let-7c-5p, miR-106a-5p, miR-20a-5p, miR-21-5p, miR-93-5p, miR-125a-5p, miR-106b-5p, miR-19a-3p, miR-15a-5p, miR-107, miR-16-5p, miR-15b-5p, miR-130a-3p, miR-301a-3p, miR-199a-5p, miR-374a-5p, miR-142-3p, miR-197-3p, miR-18a-5p, miR-23b-3p, miR-23a-3p, miR-374b-5p, miR-432-5p	BRAF, STAT3, NFKB1, SOS2, NRAS, RUNX1, PIK3CB, ZBTB16, PIK3R5, TCF7L1, RAF1, CHUK, KRAS, RPS6KB2, CCNA1, IKBKB, RUNX1T1, PIK3CD, PIK3R3, CCND1, AKT1, CEBPA, MYC, PIM1, KIT, PIK3R1, SOS1, FLT3, AKT3, PIK3CA, LEF1, MAP2K1, MTOR, RELA, TCF7, MAPK1, GRB2, RPS6KB1	0.036473
**Inflammatory mediator regulation of TRP channels**	miR-27b-3p, miR-361-5p, miR-432-5p, miR-766-3p, miR-324-5p, miR-26a-5p, miR-340-5p, miR-19a-3p, miR-130a-3p, miR-301a-3p, miR-142-3p, miR-30b-5p, miR-144-3p, miR-23b-3p, miR-23a-3p, miR-15a-5p, miR-107, miR-16-5p, miR-15b-5p, miR-21-5p, let-7a-5p, let-7i-5p, let-7d-5p, let-7f-5p, miR-98-5p, let-7g-5p, let-7e-5p, miR-106a-5p, let-7b-5p, miR-199a-3p, let-7c-5p, miR-20a-5p, miR-106b-5p, miR-18a-5p, miR-185-5p, miR-28-5p, miR-197-3p, miR-125a-5p, miR-374a-5p, miR-374b-5p, miR-24-3p, miR-93-5p	CAMK2D, PRKCA, IL1RAP, PRKCQ, ADCY1, PTGER4, IL1R1, ASIC1, ADCY7, ADCY2, CALM3, CALM1, PIK3CB, TRPM8, ASIC4, PIK3R5, PPP1CC, MAPK14, BDKRB2, PLA2G4F, MAP2K3, PRKCD, CALM2, PRKCE, TRPA1, MAP2K6, TRPV3, PLCB1, MAPK9, PIK3CD, PIK3R3, MAPK8, NTRK1, PTGER2, PRKCG, ITPR1, PIK3R1, PRKX, PLA2G4A, NGF, PRKCB, IGF1, GNAQ, MAPK12, CAMK2B, PIK3CA, F2RL1, PRKCH, HTR2C, PLA2G4E, ITPR3, ITPR2, ADCY9, ADCY4, PLCB4, PLCB2, PLA2G4C, MAPK10, PRKACB, HTR2A, PPP1CB, ADCY6	0.038189

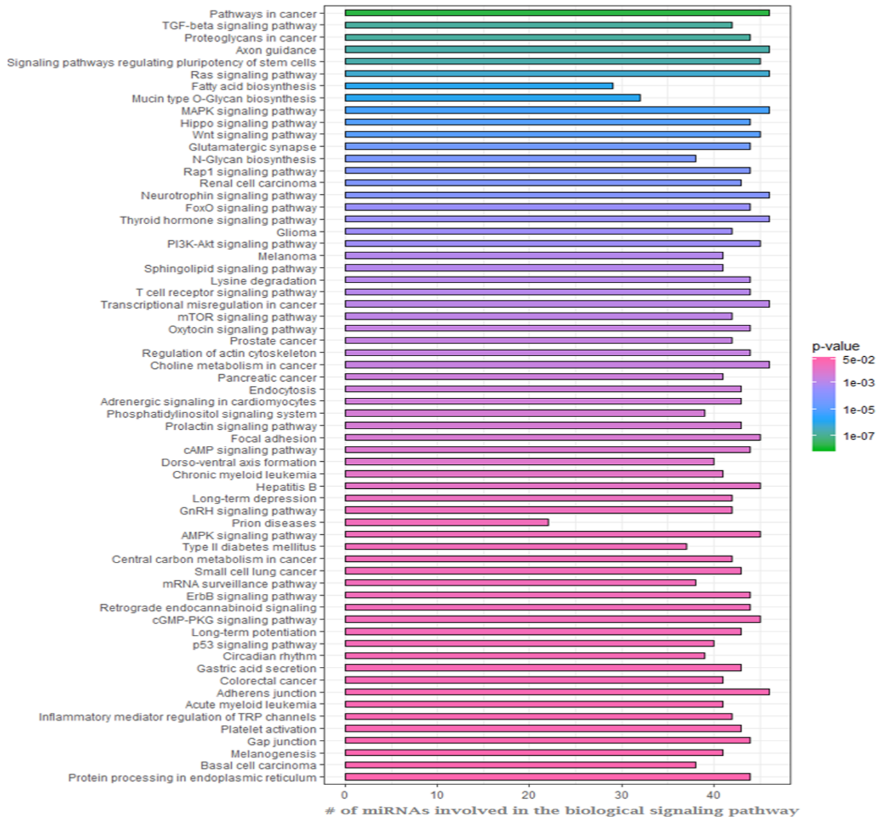

Figure S1. Full list of enriched Kegg signaling pathways for the downregulated miRNAs represented in Figure 2 ordered by their P-values (low to high). Signaling pathway were identified by the web-based computational tool DIANA-miRPath [50].

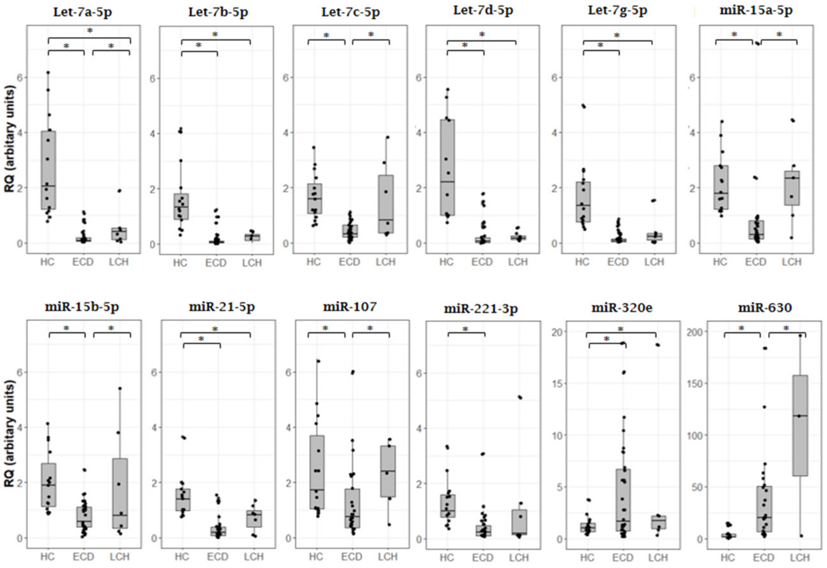

Figure S2. Validation of miRNAs by qRT-PCR. MiRNA expression in ECD and LCH patients' plasma samples compared to plasma samples from healthy control (HC). MiRNA expression was normalized to spike-in control cel-miR-39. * $p < 0.05$. Due to the lack of biological material miR-630 was evaluated in 3 LCH plasma samples and not 7 plasma samples. RQ; relative quantification.

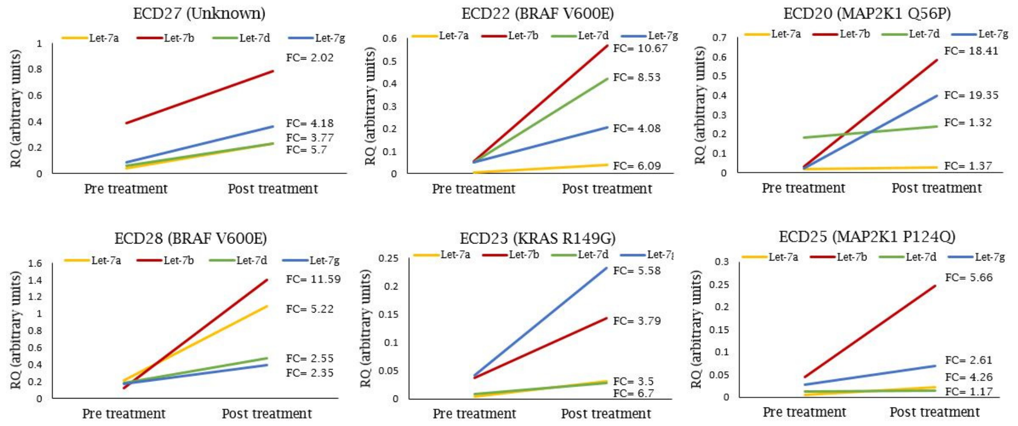

Figure S3. MiRNA expression before and after treatment with MAPK pathways inhibitors (cobimetinib and vemurafenib). The graph shows the up regulation of let-7a, let-7b, let-7d and let-7g after treatment with MEK inhibitor for 16 weeks. MiRNA expression was measured by qRT-PCR, normalized to spike-in control cel-miR-39. Fold change is shown on the right side of the line. RQ; relative quantification.

© 2020 by the authors. Licensee MDPI, Basel, Switzerland. This article is an open access article distributed under the terms and conditions of the Creative Commons Attribution (CC BY) license (http://creativecommons.org/licenses/by/4.0/).
